# Scientific collaboration of Cuban researchers working in Europe: understanding relations between origin and destination countries

**DOI:** 10.1007/s11192-018-2888-2

**Published:** 2018-08-20

**Authors:** Miriam Palacios-Callender, Stephen A. Roberts

**Affiliations:** 1 School of Computing and Engineering, University of West London, St Mary’s Road, Ealing, London, W5 5RF UK; 2 Cardiovascular Division, Faculty of Life Sciences and Medicine, King’s College London, The James Black Centre, 125 Coldharbour Lane, London, SE5 9NU UK

**Keywords:** Bibliometric study, Cuban scientists in Europe, Developing countries, Global science, Internationalization of higher education, Transnational knowledge networks, 01A90, O33

## Abstract

The global network of scientific collaboration created by researchers opens new opportunities for developing countries to engage in the process of knowledge creation historically lead by institutions in the developed world. The results discussed here explore how Cubans working in European science and technology might contribute to extending the scientific collaboration of the country through their ties with Cuban institutions mainly in the academic sector. A bibliometric method was used to explore the pattern of collaboration of Cuban researchers in Europe using the institutional affiliation of authors and collaborators. The records of scientific publications of the defined sample were obtained from *Scopus* database for the period between 1995 and 2014. The network of collaboration was generated using the affiliations of Cuban authors in Europe and co-authors with worldwide affiliations shown in the records of publications of each Cuban researcher of the study. The analysis of aggregate values of the output of Cuban researchers in Europe (1995–2014) reveals that their collaboration with Cuba correlates moderately with their performance in Europe. However, when taking into account their time publishing in Europe, the collaboration with Cuba decreases the longer they remain away from home. The network of collaborating Cuban researchers in Europe comprises 991 different affiliations from 58 countries: 698 from Europe, 118 from North America, 96 from Latin America and 79 from the rest of the world. *K*-core analysis of centrality shows two Cuban universities sharing the central position with another 24 institutions worldwide of which 18 belong to higher education.

**Electronic supplementary material:**

The online version of this article (10.1007/s11192-018-2888-2) contains supplementary material, which is available to authorized users.

## Introduction

The global network of scientific collaboration created by researchers opens new opportunities to developing countries to engage in the process of knowledge creation, historically lead by institutions in the developed world. The results discussed here explore how Cubans working in European institutions of science and technology could contribute to diversify and extend the scientific collaboration of Cuba. Developing countries whose scientists are highly mobile can benefit from an *Observatory* of their researchers performance abroad in order to formulate national science policy aiming to harness their potential. Tailoring and updating policies in both origin and destination countries can reduce the impact of losing talents, as well as benefiting all stakeholders.

Internationalization of higher education (HE) has allowed developing countries to engage in the global network of scientific collaboration by sending doctoral students to further their education in universities of the developed world, as well as exchanging students within the developing countries. However, the beneficial outcome generated by the mobility of researchers within the context of global networks of scientific collaboration turns detrimental when mobility ends in permanent migration. Their return to the country of origin still remains poor in the developing world. Two main factors affect this problem: structural, economic and financial difficulties existing in many developing countries and the opportunities offered by developed countries to retain the best and brightest researchers for the benefit of their economies (Kuptsch 2006; Docquier and Machado 2016).

Our study starts from a period in which Cuba underwent serious economic difficulties prompting some economic migration of professionals post 1990 (Casaña 2007). The economic migration was not unique to Cuba, as other countries in the Caribbean region also experienced a similar trend (Zong and Batalova 2018). Simultaneously, a younger generation of Cubans was taking part in international post-graduates programmes offered as part of the internationalization of the higher education worldwide (Hernández Pérez 2005). Under those circumstances, a sizeable sample of researchers working in European science and technology leads us to explore their nexus with the country of origin.

Cuba’s education and scientific development were priorities of the socialist government since 1959, increasing consistently the number of tertiary educated graduates since then. In 2010 there were 900,000 university graduates (Clark Arxer 2010) and between 2011 and to 2015 another 333,424 graduated from Cuban institutions of HE (see the website of the Ibero-American network on science and technology indicators, RICYT, http://www.ricyt.org/indicadores^1^). Since 1960 Cuban universities became key players in building the emerging national science sector, which turned decisive in the country’s economic, commercial and financial crisis after the collapse of socialist countries in Europe (Pérez-Ones and Núñez Jover 2009), worsened by the United States blockade on Cuba. The government effort on boosting science for the development of the country focused on re-organizing the national system with the creation in 1994 of the new Ministry of Science, Technology and Environment (Clark Arxer 2010). It has been recognized that excelling in the biotechnology sector proved to be the path in strengthening the scientific capacity of the country in response to the needs of the Cuban population (Singer and Daar 2001; Lage 2008; Lemarchand 2015, 83). However, in 2002 Cuba with 544 researchers per million inhabitants was in the fourth place in Latin America and the Caribbean region and by 2013 the country dropped to sixth place with 397 researchers per million inhabitants (UNESCO 2010, 2015). An overview and prospects on Cuban science, technology and innovation policy between 1990 and 2010 indicates the need of new policy (Nuñez Jover and Montalvo Arriete 2015). One of the topics discussed by the authors referred to strengthening the formation of human resources in careers of sciences and technology and implementing measurement for their retention. Our research offers a view about the performance of a sample of Cuban professionals of science and technology who decided to continue their careers abroad, given the economic circumstances in this period and the global trend in internationalization of the HE (Bhandari and Blumenthal 2011).

In a previous study we compared the performance of two samples of Cuban researchers in Europe (CRiE) and Cuban researchers in Cuba (CRiC). The total numbers of publications regardless of their affiliations were 7130 and 2467 articles for CRiC and CRiE respectively since they published for the first time (supplemented information in Appendix D and in Palacios-Callender 2016). The results showed that seniority (measured as the time since the author published the first article) and productivity (total number publications since the first published article) were skewed by age- and institution-related factors. In addition, CRiC generally published more in Cuban journals (Chinchilla-Rodríguez et al. 2015) with less than 12% coverage in Scopus,^2^ while CRiE published in European and International journals. The latter and the wider distribution of CRiE working in 115 European institutions compared to 14 Cuban institutions where CRiC work, pointed to new opportunities of scientific collaboration between Cuba and Europe.

In this study we aim to understand how scientific communication operates between destination and origin countries and to find the institutions leading the nexus between the global networks of scientific collaboration and local scientific output. This study will provide evidence for policy makers in both Cuba and destination countries in Europe to maximize the benefit of the interconnected world of science (Wagner and Leydesdorff 2005) and to work towards a more balanced global economy in terms of social and human development.

Two bibliometric approaches relevant to our aims have been used in high emigrating countries in order to evaluate the impact of their scientific diasporas. One study looked at a sample of scientific diaspora of Mexico, a country of the Latin America region with a big population (Marmolejo-Leyva et al. 2015) and the other, examined the situation in the small countries in the Pacific (Gibson and Mckenzie 2014). In the case of Mexico, the authors obtained the sample of scientific diaspora and the cohort group at home from the Mexican National System of Researchers (SNI). In the small countries of the Pacific, the authors selected a group of individuals who were the best and brightest at secondary education before the migration occurred identifying *a posteriori*, those researchers who migrate or stayed at home. In both cases, the authors pointed at the high productivity of those young researchers abroad compared to the cohort group at home, while retaining their ties with the home country through international collaboration, or providing research knowledge transfer by those returning home. The source of data used for the case of Mexico was *Web of Science* (*WoS*) and *Scopus* and in the case of small countries of the Pacific, *Google Scholar*.

The present study used bibliometric data to address the nexus between Cuban researchers in Europe and research institutions in their home country. The rationale underpinning the design takes into account first, that scientific publications are validated output of knowledge production and second, that the institutional affiliations of authors recorded in the databases of scientific bibliographies such as *Scopus* and *Web of Science* are tools for tracing the movement of active researchers (Plume 2012a, b; Moed et al. 2013).

One of the reasons for selecting Europe as a destination region in this study is based on the long-term cooperation in higher education and scientific collaboration of Europe with Latin American and the Caribbean countries (Gaillard and Arvanitis 2013). Scientific publications between Cuba and Europe have steadily increased since 1990 accounting for more than 55% of Cuban international collaboration (Palacios-Callender et al. 2016) in 2010. This pattern is also observed in the majority of Latin American countries collaborating with Europe (Russell and Ainsworth 2013). In a search carried out in 2018^3^ for this study, we found that the number of documents (articles only) in *Scopus* by Cuban institutions was 25,451 between 1995 and 2014, of which 38% were with international co-authorship. Those articles with European affiliation represent 56% of all international co-authored articles.

## Methodology

Essentially this study explores the performance of a sample of Cuban researchers in Europe and their patterns of scientific collaboration by analysing the affiliations of their collaborators listed in each publication from the year in which they published systematically with an address in Europe. Two approaches were used modelling the nexus between origin and destination countries aiming to establish the benefits (or disadvantages) of the scientific collaboration for the parts involved. The first approach looks at variables related to the nexus between origin and destination using aggregate values of scientific publications in the period between 1995 and 2014. The second takes into account the time when Cuban researchers began to publish systematically with European affiliations. Additionally, the analysis of the network of scientific collaboration at institutional level was also carried out in order to identify which institutions and sectors were leading the flow of knowledge through their international collaboration.

### Sample of Cuban researchers in Europe (CRiE)

There is no data available referring to the number of Cuban scientists in Europe, or a list of those remaining as active researchers. Cuban scientists abroad (diaspora) are not included in the national system of researchers. The estimated number of 10,600 Cubans in Europe with higher education was obtained by triangulation of the data given in 2007 by official Cuban sources (Martín Fernández et al. 2007) and the database using OECD sources for 2000 and 2010 (Docquier and Marfouk 2006; Brücker et al. 2013). The proportion of researchers in the field of science and engineering was roughly assumed to be between 600 and 1200 between 1995 and 2014, from the beginning to the end of the period of study. This range comes from the assumption that opportunities for research in Europe where the Cubans work, were similar to those in the United States in which the number reported of Cuban-born scientists and engineers was 64,000^4^ (Kannankutty and Burrelli 2007) out of 349,878 with tertiary education (Brücker et al. 2013). The estimation also takes into account that only 31% of immigrant scientists in the U.S. were directly involved in an occupation of science and engineering (Palacios-Callender 2016). Therefore, in the present study we aimed to reach a sample of at least one hundred active Cuban researchers in Europe (CRiE), which is in the order of 10% of those Cubans working in European institutions of science and technology previously estimated for this period.

The preliminary list of CRiE was created using methodologies applied for studying hard-to-reach populations like snowball and respondent-driven sampling, also called chain-referral sampling (Heckathorn 2011). The first names collected from the snowball technique became the starting point for a second round of chain referral sampling using professional networks such as *LinkedIn* and *ResearchGate*. Researchers included in the list were verified of having finished their HE in Cuba and that they were working in Europe. This information was taken from professional networks and *curriculum vitae* available on the Internet.

### Datasets for tracing movement and type of collaboration of CRiE

**Fig. 1:**
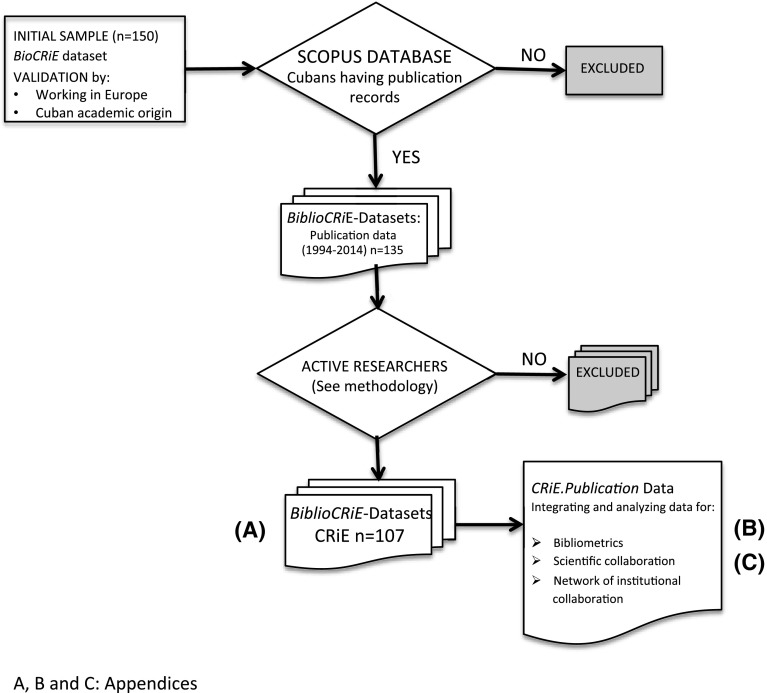
Diagram of the experimental design used in the study of Cuban Researchers in Europe (CRIE). An example (researcher 209-01) of the working dataset of 107 CRiE (BiblioCRiE of active researchers) is shown in Appendix A (A). The bibliographic information of each CRiE is analyzed and classified according to the aims of the study (see Methodology). Appendix B: Aggregate tabulation of CRiE nexus with Cuban institutions. Appendix C shows the institutional collaboration of CRiE case 209-01 (Appendix A) and how the data is prepared for the symmetric matrix (Marcet García et al. 2016) for the network analysis of scientific collaboration

The bibliographic database of *Scopus* was used to classify those Cubans by their main attribute of publishing in peer-reviewed scientific journals and finding their unique identification number (ID). Those without an ID in *Scopus* were excluded from the sample and a filter for active researcher was also applied (Plume 2012a, b; Moed et al. 2013) counting only <articles> as documents of interest. The search in *Scopus* was carried out through the engine <author search> using alternative entrance with the two surnames traditionally used in Hispanic culture. The publication records of each researcher with *all available information* was downloaded and codified anonymously. Only researchers publishing at least 10–15 articles in the period (1995–2014) and at least once in the last 5 years (remaining as active researchers) were included. Newcomers with less than 10 articles in the period, but at least two articles in the last 5 years were also included. Two main datasets were created for the study: *BioCRiE*, with information gathered from public sources including curriculum vitae (when it was available) and *BiblioCRiE* with *Scopus* information about their publications (Appendix A, one example: BiblioCRiE 209-01). A diagram of the methodology is shown in Fig. 1. Files were anonymized and codified. The code assigned to each CRiE refers to the country where the researcher was working at the time it was included in the list of CRiE.

Disambiguation of affiliations were carried out manually using additional information on the Internet about the place, mainly the official website of the institution. University hospitals were considered part of the main university. Whenever applicable, authors working in units of the European national institutions for research within the academia received the affiliation of the university in question.^5^ Collaborators with two independent affiliations in the same article were counted as two. In the case of a CRiE with two independent affiliations, the second address is treated as collaboration with, but if the Cuban affiliation was the main address, the article was counted as research experience in the country of origin (*P*_C_, see definition in the next section *nexus with the country of origin*).

Articles per researcher were classified according to participating institutions: only one national institution (N), more than one national institution (NN), only one national institution with one or more international institutions (NI) and more than one national institution with one or more international institutions (NNI) as seen in Appendix A (BiblioCRiE 209-01). National refers to the country in Europe from which the author is publishing.

### Nexus with the country of origin and network of scientific collaboration

To explore the nexus between CRiE and Cuban scientific institutions their papers were inspected for Cuban addresses shown in each publication between 1995 and 2014. For each CRiE the number of articles published using Cuban affiliations was counted and considered as *CRiE* with research experience in the country of origin (*P*_C_). Likewise, the number of articles per CRiE published using European affiliation (*P*_E_) was counted. The time from which a Cuban researcher is considered a CRiE takes into account not only when the affiliation is in Europe, but also the records of their job positions in Cuba and abroad shown in *LinkedIn*, or in available curriculum vitae (some cases the information was obtained contacting the researcher by email).

To measure their ability for collaboration the number of collaborative articles per CRiE from European affiliations was also counted (*C*_E_: NN + NI + NNI) identifying and counting the number of articles in collaboration with Cuban institutions (*C*_C_). Therefore, each CRiE has four variables associated: *P*_C_, *P*_E_, *C*_E_ and *C*_C_ (see Appendix B CRiE nexus Cuba created from processing data of 107 BiblioCRiE). Pearson’s correlation was used to analyse the relation between the variables involved in the model.

The network of scientific collaboration of CRiE was analysed by creating a symmetrical matrix with the institutions shown in the field of author’s affiliation. Each European institution from which CRiE were publishing was considered as a type I institution and those collaborating institutions from the rest of the world were called type II institutions in order to create a binary relational matrix to explore institution-to-institution collaboration (Phillips et al. 2013). Links between type I (*x*) and type II (*y*) institutions were obtained from the publications of 107 CRiE in aggregate between 1995 and 2014. Each research collaboration with type II institutions was counted as an integer counting. In the case of more than one collaborator with the same affiliation (even from different departments, or particular addresses of the same institution) the link was counted as one. The three columns representing type I, type II and the number of links of collaborations (See fragment in Appendix C: *addresses* and *matrix* from Appendix A, BiblioCRiE 209-01) were transformed in a symmetrical matrix using an ad hoc programme (Marcet García et al. 2016) and then processed through UCINET and NetDraw software for the *k*-core analysis and visualization of the institutional network of collaboration (Borgatti et al. 2013).

## Findings

### Validation of the sample of Cuban researchers in Europe

The approach used to create the list of Cuban researchers in Europe yielded 90% of Cubans showing an ID in *Scopus* (135/150), of which 78.5% were active researchers. Two main reasons might explain the high yield of Cuban professionals of S&E with records in *Scopus* as well as the proportion classified as active researchers. First, the use of professional networks, such as *LinkedIn* and *ResearchGate* to find the individuals of the sample, and second the process used for disambiguation of names/surnames of each individual. Professionals interested in developing their careers are active members in the professional networks sharing contacts, interests and achievements with their peers. In terms of analysing the number and frequency of publications per researcher, disambiguation was vital to avoid split identities. Indeed, we found 28 researchers with two or three IDs in *Scopus*, including three cases due to changing surnames after marriage.

Another characteristic of CRiE was their mobility within European institutions: those with more than one affiliation represent 46%, of which 63% involved the movement to at least another country, including the United States and Latin America, but returning to European affiliations. This might be another factor behind multiple identities (*Scopus* IDs). CRiE have been publishing from 115 European institutions of which 80 were universities, 28 national or regional institutes of research and 7 institutions belonged to the industry.

### Pattern of scientific publications of CRiE

One hundred and seven active Cuban researchers in Europe (CRiE) published 2385 scientific articles of which 1863 were with European affiliations during the period between 1995 and 2014 (Table 1). Their publications with European affiliations were more than twice the volume of those published with Cuban affiliations. Eight CRiE published twenty-seven articles with Cuban addresses before 1995 (these publications were not included in this study), of which seven moved to Europe between 1996 and 2001 and one in 2006.

**Table 1:** Productivity and scientific collaboration of CRiE by country of the last affiliation

Country	Number CRiE	Publication (1995–2014)			Type of publication by participating institutions					
		Total	In Europe	In Cuba	In Europe				in Cuba	
					N	NN	NI	NNI	N	NN + NI + NNI
Belgium	11	231	199	32	68	23	74	34	5	27
Denmark	1	3	2	1	0	1	1	0	0	1
Finland	3	63	56	7	9	15	21	11	1	6
France	6	91	39	52	4	10	19	6	6	46
Germany	12	179	145	34	33	27	41	44	11	23
Italy	10	105	80	25	40	21	16	3	9	16
Luxemburg	1	15	13	2	3	5	2	3	0	2
Netherlands	1	14	12	2	2	1	9	0	1	1
Portugal	1	31	21	10	0	0	21	0	0	10
Spain	36	848	627	221	197	171	160	99	47	174
Sweden	4	133	101	32	8	1	84	8	4	28
Switzerland	4	44	30	14	16	3	8	3	8	6
UK	17	628	538	90	135	72	162	169	30	60
Total	107	2385	1863	522	515	350	618	380	122	400

Number of Cuban active researchers in Europe (CRiE) and their publications between 1995 and 2014 by country of affiliation shown in *Scopus*. Publications were classified according to their collaboration: non-collaborative (N), those collaborating with another national institution (NN), or with one or more international institutions (NI) and with national and international institutions (NNI)

The analysis per destination country is not valid due to the mobility of researchers in the region. For instance, one CRiE counted in the UK because this is her current address in *Scopus*, published 56 articles with a French affiliation and 137 with a British affiliation. Then, by not counting the 56 articles within the French records leads to assume that researchers working in France have published on average less. Other factors affecting the analysis per country are the number of years spent by CRiE in Europe, the number of researchers per country and their field of research. Nevertheless, it is clear that CRiE are highly collaborative researchers with 72% of their scientific output in collaboration with other institutions, of which 74% were international collaboration (NI + NNI) and 54% were national collaboration (NN + NNI). This pattern was also observed when CRiE were publishing using Cuban affiliations, having 77% of collaborative papers (NN + NI + NNI, see Table 1).

### Nexus with the country of origin and network of scientific collaboration

Given the collaborative pattern of CRiE we wanted to know if having experience in Cuban institutions might contribute to further collaboration between European institutions and Cuba. The sample of CRiE were classified into four groups according to their values of *P*_C_, *P*_E_, *C*_E_ and *C*_C_ previously described in methods:

- P1C1: *Researchers with experience in Cuba* (*P*_C_ > 0) *and collaborating with Cuba* (*C*_C_ > 0)
- P1C2: *Researchers with experience in Cuba* (*P*_C_ > 0) *and without collaboration with Cuba* (*C*_C_ = 0)
- P2C1: *Researchers with no experience in Cuba* (*P*_C_ = 0) *and collaborating with Cuba* (*C*_C_ > 0)
- P2C2: *Researchers with no experience in Cuba* (*P*_C_ = 0) *and without collaboration with Cuba* (*C*_C_ = 0)

The characteristic of the experimental design considering the outward movement of Cuban researchers towards Europe (Fig. 1) implies that their publications with Cuban affiliations (*P*_C_) preceded their publications and collaboration in Europe (*P*_E_, *C*_E_ and *C*_C_). A manual inspection of the chronological records of publishing showed that 19 CRiE out of 107 have published 41 articles using Cuban affiliations while in Europe (*P*_C_), of which 17 have collaborated with Cuba subsequently. Additionally 16 CRiE have published 64 articles using Cuban addresses as a second affiliation, which represented the 31.5% of CRiE collaboration with Cuba (*C*_C_). Only six CRiE showed both conditions indicating that 29 CRiE (27%) experienced transnational knowledge practices after using regularly the European affiliation. We followed the researchers by their publishing pattern and not by their migration status. Temporary visas and naturalization options vary among countries in Europe. Moreover, the free mobility within the European Union opens jobs opportunities for researchers from third countries to stay. On the other hand, modification of the Cuban migration law in recent years might help mobility over migration^6^ for those Cuban researchers abroad.

**Fig. 2:**
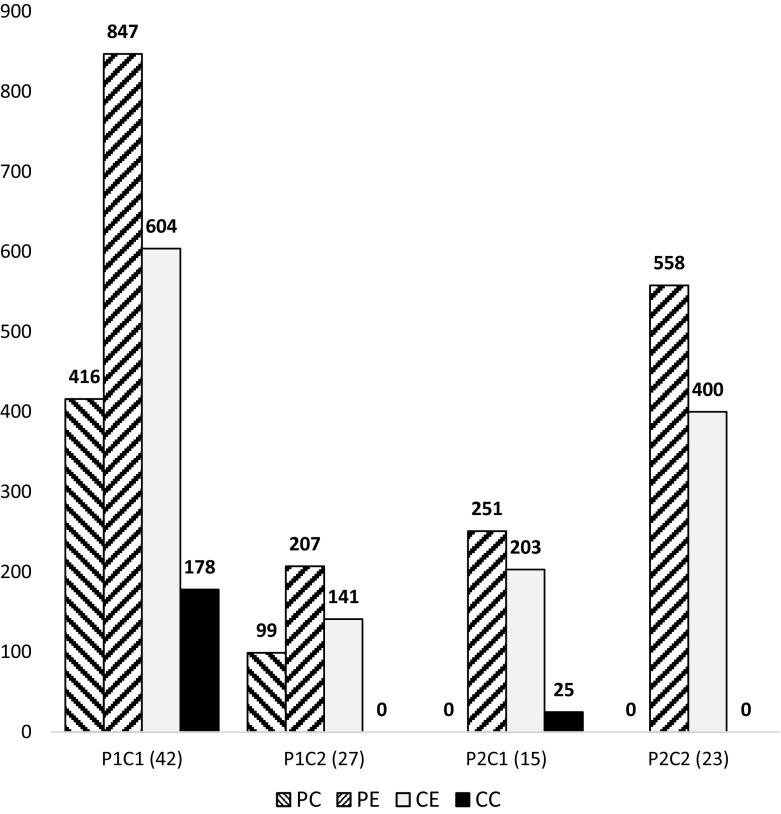
Output of Cuban researchers in Europe according to their nexus with the country of origin. Aggregate publications and collaboration of CRiE in groups C1P1, C1P2, C2P1 and C2P2 (see Methodology) with Cuban (*P*_C_) and European (*P*_E_) affiliations and total number of collaboration from Europe (*C*_E_) of which (*C*_C_) were with Cuban institutions are shown in columns. In brackets number of researchers per group

Figure 2 shows the aggregate values of publications with Cuban affiliation (*P*_C_), and European affiliations of CRiE (*P*_E_), as well as the number of collaborative articles with their new affiliation in Europe (*C*_E_) and how many of them were with Cuban institutions (*C*_C_). P1C1 is the group with more researchers (42) followed by P1C2 (27) indicating that 65% of CRiE had previous experience in Cuban institutions of S&E. CRiE without collaboration with the country of origin but with previous experience in Cuba (P1C2) seem to be less productive as the mean values for *P*_C_ and *P*_E_, are less than half of those in the group P1C1 (3.7, 7.7 vs. 9.7, 20.1), Table 2. P1C2 seems less inclined to institutional collaboration with other institutions by having a mean *C*_E_ of 5.2 collaborative articles, while P1C1, P2C1 and P2C2 mean values of *C*_E_ were 14.5, 14.5 and 17.4 respectively. Summarized data for *P*_C_, *P*_E_, *C*_E_ and *C*_C_ from 107 BiblioCRiE are in Appendix B (CRiE nexus Cuba).

**Table 2:** Group of researchers according to their nexus with Cuba

Variables	Groups	P1C1	P1C2	P2C1	P2C2	P*i*C*i*
	*n*	42	27	15	23	107
*P*_C_	SUM	416	99	0	0	515
	Mean	9.7	3.7	0	0	4.8
	SD	13.6	2.8	0	0	9.7
*P*_E_	SUM	847	207	251	558	1863
	Mean	20.1	7.7	18.3	24.3	17.4
	SD	28.4	7.0	17.7	42.7	27.9
*C*_E_	SUM	604	141	203	400	1348
	Mean	14.5	5.2	14.4	17.4	12.6
	SD	18.9	5.4	16.1	36.4	21.7
*C*_C_	SUM	178	0	25	0	203
	Mean	4.3	0	1.7	0	1.9
	SD	6.3	0	0.9	0	4.4

Columns representing groups as P1C1: 42 *researchers with experience in Cuba* (*P*_C_ > 0) *and collaborating with Cuba* (*C*_C_ > 0); P1C2: 27 *researchers with experience in Cuba* (*P*_C_ > 0) *and without collaboration with Cuba* (*C*_C_ = 0); P2C1: 15 *researchers with no experience in Cuba* (*P*_C_ = 0) *and collaborating with Cuba* (*C*_C_ > 0); P2C2: 23 *researchers with no experience in Cuba* (*P*_C_ = 0) *and without collaboration with Cuba* (*C*_C_ = 0). P*i*C*i* represents all CRiE with *n* = 107 researchers. Publications by CRiE signing with Cuban (*P*_C_) and European (*P*_E_) affiliations and total number of collaboration from Europe (*C*_E_) of which (*C*_C_) were with Cuban institutions

The Pearson’s correlation coefficients shown in Table 3 indicate the relations between variables *P*_C_, *P*_E_, *C*_E_ and *C*_C_ for the whole sample (P*i*C*i* = 107) and for different sub-groups regarding the CRiE nexus with Cuba. The result analysing the sample of 107 CRiE shows that only *P*_E_ and *C*_E_ have a positive and a strong correlation (*r* = 0.965), while the correlation of *P*_E_ and *C*_E_ with the ability to collaborate with Cuba (*C*_C_) is moderate (0.412 and 0.407 respectively). Publishing from Cuban institutions (*P*_C_) has a positive and a moderate correlation with the ability of CRiE to collaborate with Cuba (*r* = 0.329). It seems that their experience publishing from Cuba (*P*_C_) has almost no correlation with CRiE performance in Europe (*P*_E_ and *C*_E_) with Pearson’s correlation coefficients of 0.008 and 0.057 respectively. Interestingly, when expecting stronger correlation between *P*_C_ and *C*_C_, for the sub-group P1C1of CRiE, the Pearson’s correlation coefficient became smaller (*r* = 0.195). Instead of the research experience in Cuba (*P*_C_) having stronger correlation with home collaboration (*C*_C_), it is their performance in Europe (*P*_E_ and *C*_E_) what correlates stronger with the ability to collaborate with Cuba, having *r*-*values* of 0.661 and 0.765 respectively. This correlation of the CRiE performance in Europe (*P*_E_ and *C*_E_) with their collaborative nexus with Cuba (*C*_C_) remained strong in the sub-groups of 69 CRiE with experience publishing with Cuban affiliations (P1C1 + P1C2) and in the 55 CRiE publishing with Cuban co-authors (P1C1 + P2C1). The analysis excluding researchers without nexus with Cuba (P2C2, *n* = 83) shows the same trend (Table 3). The strongest correlation for all sub-groups was between *C*_E_ and *P*_E_ with values over 0.96 (Table 3, bold numbers). In terms of the performance of CRiE in Europe (*P*_E_ and *C*_E_), the experience in Cuba (*P*_C_) seems to be less relevant.

**Table 3:** Pearson’s correlation coefficients between variables *P*_C_, *P*_E_, *C*_C_ and *C*_E_

	*P*_C_	*P*_E_	*C*_E_	*C*_C_
P*i*C*i* (107)				
*P*_C_		0.008	0.057	0.329
*P*_E_			**0.965**	0.412
*C*_E_				0.407
*C*_C_				
P1C1 (42)				
*P*_C_		0.151	0.122	0.195
*P*_E_			**0.968**	**0.661**
*C*_E_				**0.765**
*C*_C_				
P1C1 + P2C1 (55)				
*P*_C_		0.167	0.123	0.231
*P*_E_			**0.964**	**0.628**
*C*_E_				**0.694**
*C*_C_				
P1C1 + P1C2 (69)				
*P*_C_		0.222	0.198	0.264
*P*_E_			**0.97**	**0.682**
*C*_E_				**0.773**
*C*_C_				
P*i*C*i *− P2C2 (83)				
*P*_C_		0.195	0.158	0.27
*P*_E_			**0.964**	**0.648**
*C*_E_				**0.708**
*C*_C_				

These results together with the high variability of each group (see mean and standard deviation per group Table 2) led us to analyse their output as a function of the time from when CRiE began to publish with European affiliation. For instance, the group P2C2 having the best performers, the researchers were also publishing in Europe for the longest period of time.

In general, it seems that the longer CRiE remain in Europe the less probable the collaboration with Cuban institutions (*C*_C_) is, although their overall productivity in Europe (*P*_E_) depends strongly on collaboration (*C*_E_). Taking the variables *P*_C_, *P*_E_, *C*_C_ and *C*_E_ as aggregate values for the 20 years period of study are likely to be affecting this model.

The way the sample was built, in which researchers have to be active for a period of 20 years, generated an uneven sample in terms of years of experience. Only a third of the sample have been publishing for 10–20 years, while two-thirds have been publishing for up to 10 years between 2004 and 2014. Indeed, we previously found that 72% CRiE were researchers younger than 40 years old (Palacios-Callender and Roberts 2016).

**Fig. 3:**
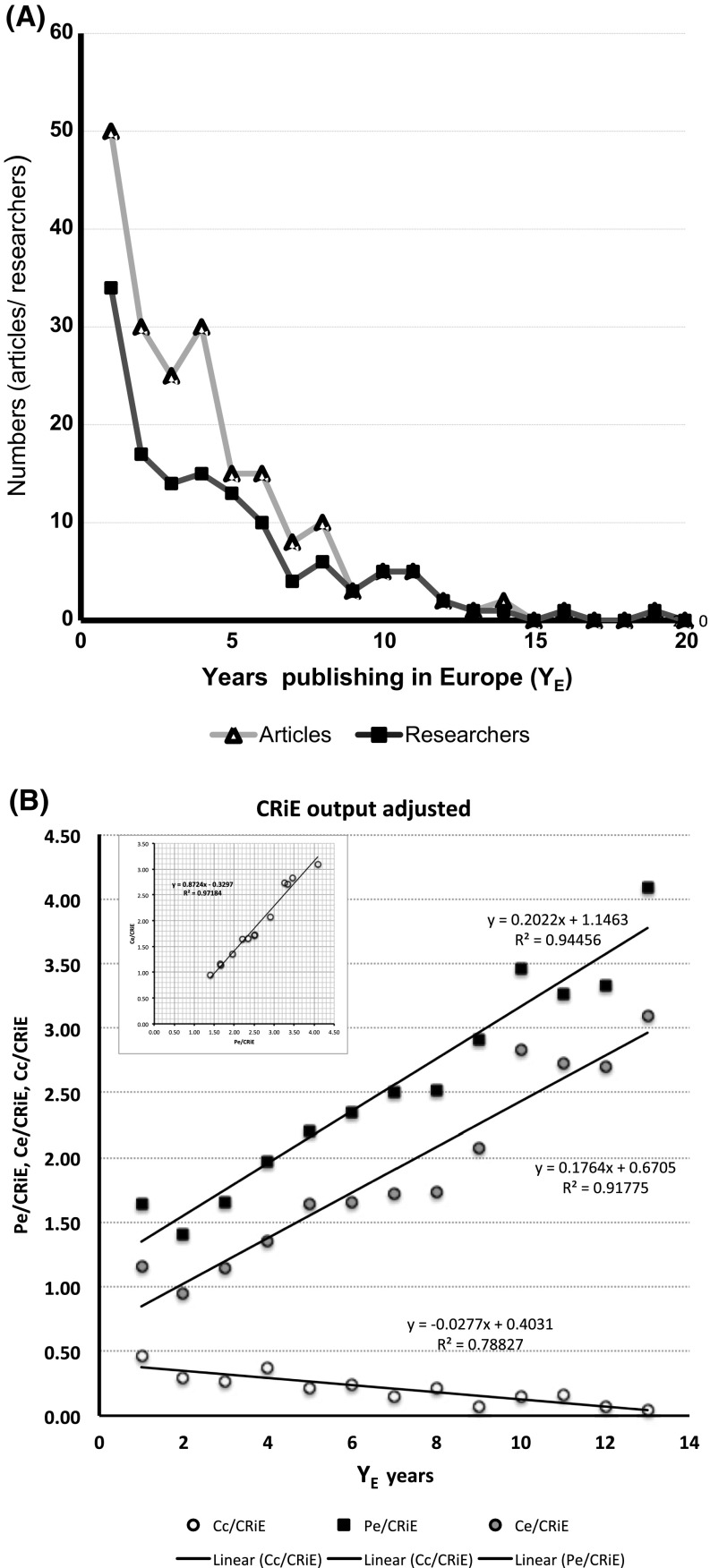
Evolution of the output of Cuban researchers in Europe. (**A**) Number of collaborative articles with Cuba starting from the year researchers moved to Europe (*Y*_E_, years publishing in Europe) and the number of those CRiE researchers involved in the collaboration with Cuba. (**B**) Scientific output (*P*_E_, *C*_E_ and *C*_C_) of CRiE normalized for the time since their first year publishing in Europe (*Y*_E_). Graphic supported by data in Table 4

To investigate further which other factor could be modifying the collaboration with Cuba, we normalized the time of their output from the first year CRiE published in Europe (*Y*_E_). Figure 3A shows the number of CRiE (only P1C1 + P2C1, *n* = 56) and their collaborative articles with Cuban institutions normalized to the year they moved to work in Europe (*Y*_E_). Thirty-four (32%) CRiE have published at least one paper in co-authorship with Cuban institutions in the first year signing with European affiliation and fifty-six (52%) have published at least once during the 20-year period of study. The number of CRiE publishing in collaboration with Cuban institutions drops after the first 5 years of publishing in Europe, while their number of publications and general collaboration increases (Table 4). Almost 75% (150/203) of the collaborative output with Cuba took place in those 5 years after moving to continue their careers in Europe, being carried out by an average of nineteen researchers per year, indicating that some of them were publishing more than one collaborative article with Cuba. We found, after adjusting to the number of CRiE publishing per year (*Y*_E_), that the share of articles in collaboration with Cuba decreases while the share of their performance (*P*_E_ and *C*_E_) in Europe increases (Fig. 3B and Table 4) overtime. The inserted graphic in Fig. 3B confirmed the observation in the previous model regarding *P*_E_ and *C*_E_ with a linear coefficient of regression of 0.97. However, this output only covers the first 13 years of CRiE publishing using European affiliations (from 2014 back to 2002). If the whole period is taken into account (2014–1995), *r*^2^ dropped to 0.67. The trends, although adjusted for the number of CRiE and their time in Europe (*Y*_E_), do not take into account the dynamic of the profession of scientists (junior PhD students, postdocs or senior team leaders). During the first 13 years of publishing with European affiliation, CRiE (81%) have generated 1705 articles (*P*_E_) and 1235 collaborative articles (*C*_E_), of which 16% were in collaboration with Cuba (*C*_C_: 199 articles). More senior CRiE (19%), who arrived before 2001 still showed 71.5% of collaborative articles (*C*_E_) of which 3.5% include Cuban affiliations (*C*_C_) after 20 or 13 years of publishing with European affiliations. Although in less proportion, the ties with the country of origin have remained almost through the 20 years working in Europe (see Table 4).

**Table 4:** Output of CRiE: *P*_E_, *C*_E_ and *C*_C_ normalized to the first year (*Y*_E_) using European affiliation as the place of work

Years (*Y*_E_)	CRiE/*Y*_E_	*P*_E_	*P*_E_/CRiE	*C*_E_	*C*_E_/CRiE	*C*_C_	*C*_C_/CRiE
1	107	176	1.64	124	1.16	50	0.47
2	101	142	1.41	96	0.95	30	0.30
3	94	156	1.66	107	1.14	25	0.27
4	82	161	1.96	111	1.35	30	0.37
5	69	152	2.20	113	1.64	15	0.22
6	61	143	2.34	101	1.66	15	0.25
7	53	133	2.51	91	1.72	8	0.15
8	48	121	2.52	83	1.73	10	0.21
9	42	122	2.90	87	2.07	3	0.07
10	35	121	3.46	99	2.83	5	0.14
11	30	98	3.27	82	2.73	5	0.17
12	27	90	3.33	73	2.70	2	0.07
13	22	90	4.09	68	3.09	1	0.05
14	20	52	2.60	35	1.75	2	0.10
15	13	25	1.92	16	1.23	0	0.00
16	11	24	2.18	20	1.82	1	0.09
17	8	20	2.50	18	2.25	0	0.00
18	5	23	4.60	19	3.80	0	0.00
19	4	5	1.25	2	0.50	1	0.25
20	2	9	4.50	3	1.50	0	0.00
		1863		1348		203	

The number of publications with European affiliations (*P*_E_) and the number of collaborative articles (*C*_E_) including collaboration with Cuban institutions (*C*_C_) were normalized to the first year using European affiliation (*Y*_E_) and adjusted to the number of CRiE in a given *Y*_E_ year. Then for *Y*_E_ = 1, 107 CRiE accrued 176 articles (*P*_E_) and 124 collaborative articles (*C*_E_), of which 50 were in collaboration with Cuban institutions (*C*_C_)

The two models explored in this study also reveal the need of classifying the researchers according to their performance (and stages of their careers) as shown in the study of Mexican diaspora using the data from the Mexican National System of Researchers (Marmolejo-Leyva et al. 2015). National database built on researchers at home and those abroad could support further assessment of the situation.

Furthermore, we looked at the institution-to-institution ties in those 1348 collaborative articles generated by CRiE to assess their degree of connectivity with the global network of scientific collaboration. The analysis of affiliations in the 1348 collaborative articles published by CRiE in Europe shows as expected, that the international collaboration within Europe dominates over other regions and countries. CRiE have collaborated with 991 institutions from 56 different countries, of which 698 were European institutions. European institutions sharing Latin roots are highly represented among institutions of the region in which Spain shares 24% of collaborative institutions, followed by Italy (15%) and France (14%). This characteristic of cultural ties in scientific collaboration was also observed in other studies (Luukkonen et al. 1993; Franzoni et al. 2012).

**Fig. 4:**
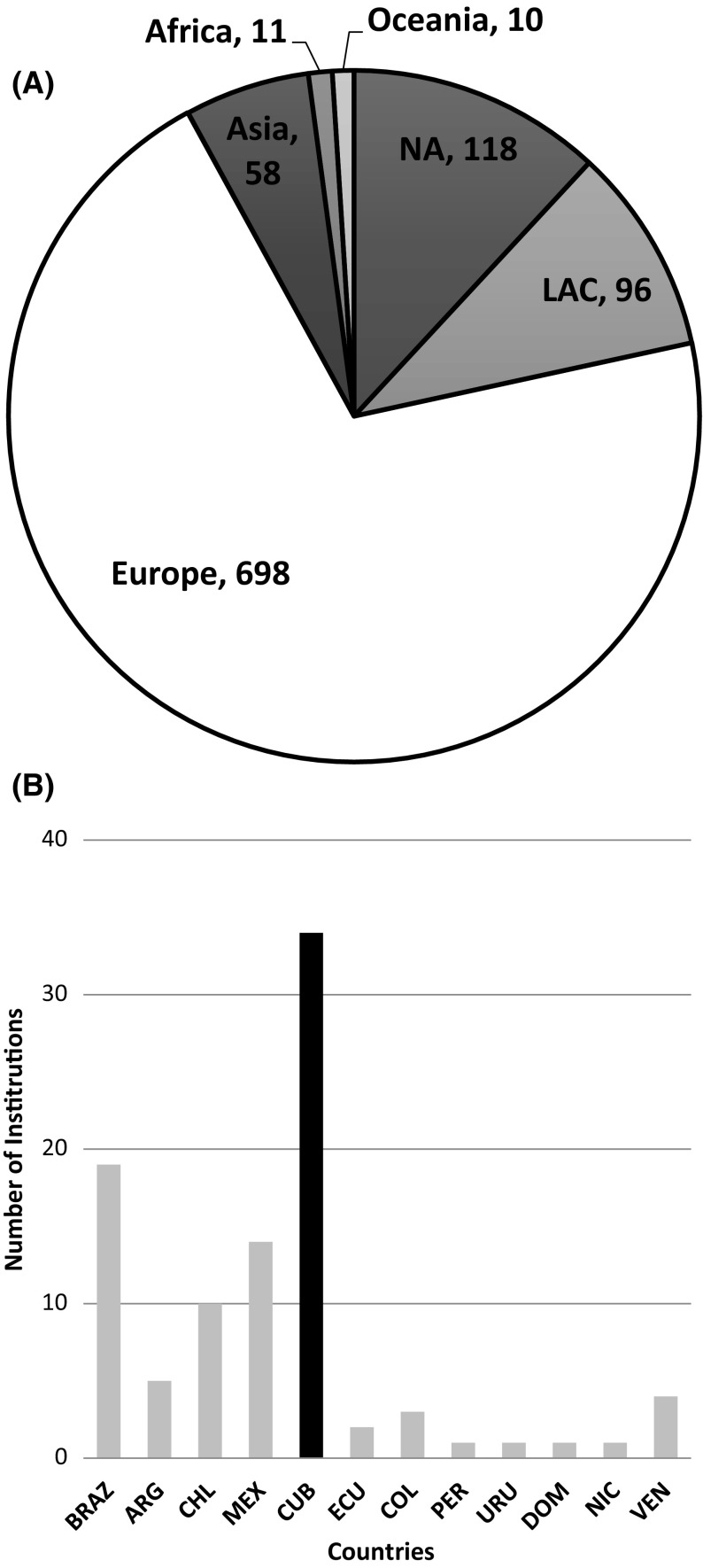
Network of institutional collaboration of CRiE (1995–2014). (**A**) Share of institutional collaboration (follow by the number) per region involved in the network of CRiE. (**B**) Number of collaborative institutions per country of Latina American and Caribbean region. Brazil (19), Argentina (5), Chile (10), Mexico (14), Cuba (35), Ecuador (2), Colombia (3), Peru (1), Uruguay (1), Dominican Republic (1), Nicaragua (1) and Venezuela (4)

Active CRiE seem to have a distinctive pattern of preferential collaboration for Latin American institutions in the Americas region taken into account the world share of publications per region. Ninety-six Latin American institutions are in the network of collaboration (45%) out of 214 institutions in the Americas, while United States and Canada are represented by 118 (55%) as shown in Fig. 4A. North America’s share of the world scientific publications was eight times bigger (31.1%) than the share of Latin America (3.6%) in 2008 (Hollanders and Soete 2010). The composition of Latin American affiliations participating in the network of CRiE’s collaboration shows (Fig. 4B) relatively more Cuban institutions compared with the rest of the region (36.5%). This proportion indicates their strong link with the country of origin when taking into consideration the country’s share of scientific publications in the region. Cuban’s shares of publication in the region have been between 1.5% in 2007 and 1% in 2014 (UNESCO 2010, 2015). Moreover, the thirty-five Cuban institutions participating in the network accrued 251 links of collaboration, followed by fourteen Mexican institutions with 33 links, nineteen institutions from Brazil with 29 links and ten from Chile with 16 links of collaboration.

**Fig. 5:**
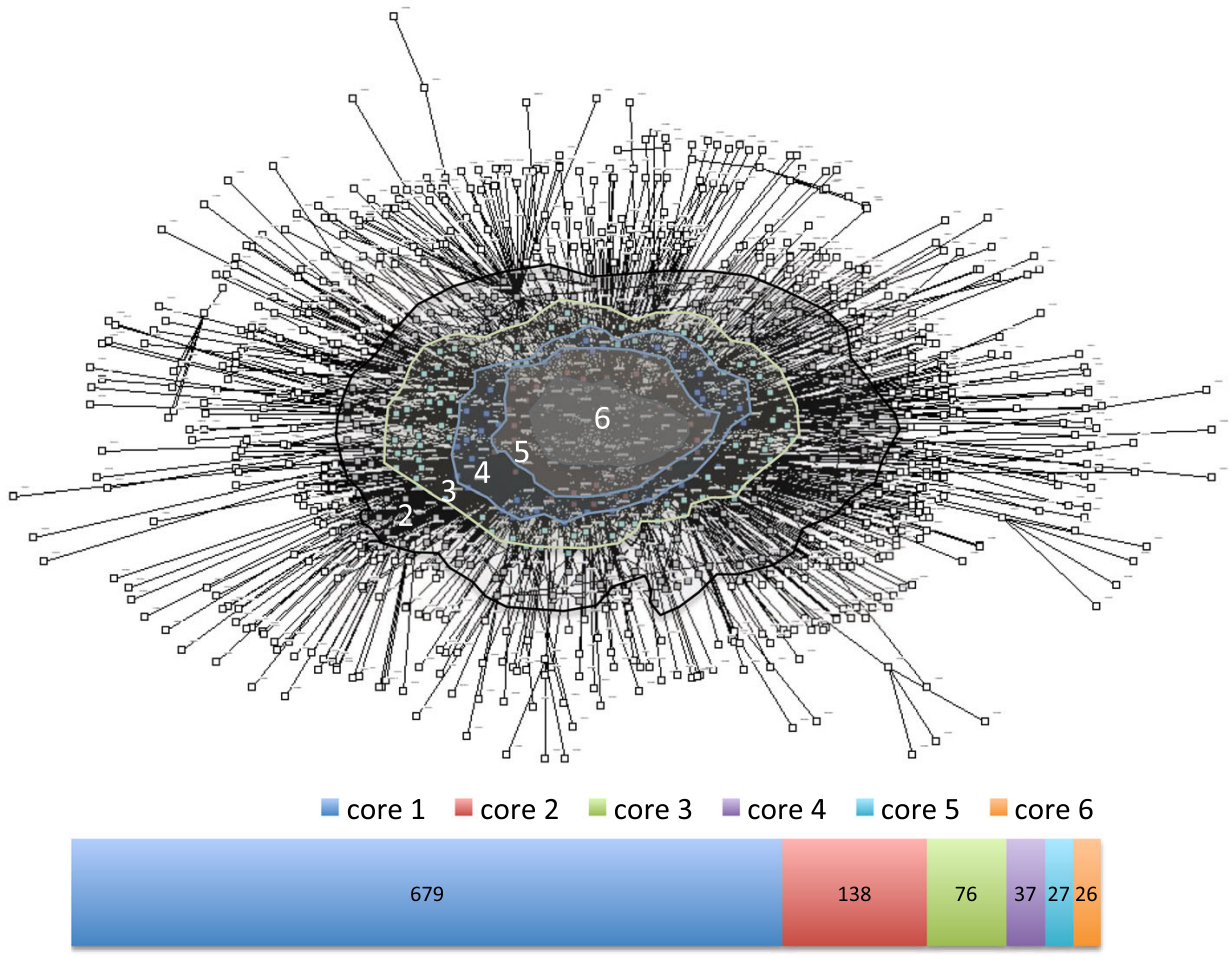
Cores and number of institutions per core in the CRiE network of scientific collaboration. Representation of the network of scientific collaboration of Cuban researchers in Europe (CRiE). *K*-core analysis was carried out using UCINET software (Borgatti et al. 2013). The bottom bar shows the number of institutions per core

The main CRiE network of scientific collaboration comprises 985 nodes (institutions in collaborative articles) and 3140 ties of collaboration accruing 6842 links or frequencies in which the collaboration took place (Fig. 5). Sub-graphics of another four European institutions with five, four and two ties respectively, are also part of the whole network of CRiE of which two belong to the industry sector. The visualization of the network shown in Fig. 5 takes into account the *k*-core analysis of centrality made using UCINET and NetDraw (Borgatti et al. 2013). At the centre of the CRiE network (*k* = 6) are 26 densely connected institutions, followed by cores *k* = 5 and *k* = 4 with another 27 institutions and 76 institutions respectively well connected. Towards the periphery with less ties connecting to other institutions, are another 138 (*k* = 3) and 679 (*k* = 2) institutions respectively (bottom bar in Fig. 5). The positions of Cuban institutions from core *k* = 6 to periphery are: 2 institutions in the core *k* = 6, one in *k* = 4, five in *k* = 3 and another five in core *k* = 2. Institutions with less than one tie are excluded in the analysis run by UCINET.

Spanish academic institutions are highly represented in the inner core (*k*-6) of the network both in number (7 Spanish institutions out of 20 academic institutions) and quality (6 Spanish institutions out of 16 academic institutions are in the top 500 of the Academic Ranking of World Universities, ARWU) as shown in Fig. 5 and Table 5. Three of these Spanish Universities are also among the top international collaborators of Cuban institutions (University of Santiago de Compostela, Complutense University of Madrid and Autonomous University of Madrid).^7^ Another institution in the central core within the top 200 universities (ARWU) is the National Autonomous University of Mexico. The UNAM is as well, among the top ten institutions collaborating with Cuba (see footnote 7). Three universities from the United Kingdom within the top 25 universities of the world (ARWU) and the first fifth place in Europe, are in the *k*-6 core, but none of them have a relevant position among international collaborators with Cuba (Table 5 and see footnote 7). One of the three German universities in the inner core of CRiE network, the Goethe University Frankfurt, is in the network of collaborators with Cuba (see footnote 7). The International Agency for Research on Cancer in Paris, France has also a central position as shown in Table 5. Two Cuban universities: University of Havana and Central University of Villa Clara are in the central position (*k*-6 core) of the CRiE network of international collaboration. These two universities are in the first and forth positions of Cuban institutions by their number of publications (see footnote 7).

**Table 5:** Institutions in the *k*-6 core of the scientific collaboration of CRiE and their AWRU ranking position

Code	Name of institutions in core *k* = 6	AWRU (2014)	ARWU (country)
201001	Catholic University of Leuven, Belgium	96	2
201002	Free University of Brussels, Belgium	301–400	3–4
202070	International Agency for Research on Cancer, Paris, France		
203013	Technical University Munich	53	3
203015	The Max Planck Institute for Coal Research, Germany		
203029	Goethe University Frankfurt, Germany	150–200	5–7
203035	Ulm University, Germany		
206002	University of Porto, Portugal	301–400	2
207001	University Santiago de Compostela, Spain	401–500	9–12
207003	University Miguel Hernandez, Spain		
207004	Health Institute Carlos III, Spain		
207012	Autonomous University Madrid (UAM), Spain	201–300	2–4
207014	Complutense University of Madrid, Spain	301–400	5–8
207017	Catalan Institution for Research and Advanced Studies (ICREA), Spain		
207020	Barcelona Autonomous University (UAB), Spain	201–300	2–4
207023	University of Barcelona, Spain	151–200	1
207024	Institute for Biomedical Research (IIB), Barcelona Spain		
207027	Spanish National Cancer Research Centre (CNIO), Spain		
207030	University Pompeo Fabra, Spain	301–400	5–8
209005	Karolinska Institute, Sweden	47	1
210003	University College London, United Kingdom	20	3
210004	University of Oxford, United Kingdom	9	1
210020	Imperial College of Science, Technology and Medicine, U.K.	22	4
210021	Wellcome Trust Sanger Institute, Cambridge, U.K.		
101003	University of Havana, Cuba		
101005	Central University of Villa Clara, Cuba		
104002	National Autonomous University of Mexico	151–200	1
301009	National Institutes of Health, United States		

*ARWU* Academic Ranking of World Universities

In the core *k* = 5, twenty-two academic institutions (79%) are contributing to knowledge flows (Fig. 5). Ninety-one percent of academic institutions are European and the majority are included in the ARWU. Among those top universities in this core are: Free University of Brussels in Belgium; University of Nice-Sofia-Antipolis, University Pierre et Marie Curie (Paris 06) and University Aix Marseille in France; University of Padua, University of Milan, and University of Turin in Italy; University of Malaga, Technical University of Catalonia, University of Zaragoza, University of Vigo and University of Laguna in Spain; University of Bristol and University of Cambridge in the United Kingdom and Harvard Medical School and University of California in the United States. However Cuban institutions are not represented here.

In the whole network of CRiE, fourteen Cuban institutions (40%) belong to the academic sector generating 74% of the collaborative links (185/251), followed by the industrial sector with 10 institutions contributing to another 15% of the collaborative links. Other sectors represented are Ministry of Science Technology and Environment (CITMA) and service sector (mainly health service).

## Discussion

A possible limitation of this study is the method used to obtain the sample of Cuban researchers working in Europe. This is not a random sample of Cuban researchers in the region for the last 20 years, but we believe it is a sufficiently representative quota of a study population as it has been in similar studies (Franzoni et al. 2012). The first constraint is that an official record with the names of researchers working abroad was not available for this study, and second by selecting active researchers for the whole period, in the way we and other authors have used before (Plume 2012a, b) we have gathered a majority of young researchers and a minority of successful researchers in the whole 20 years period (Palacios-Callender and Roberts 2016). A positive consideration to be taken in this work is the breadth of the field of research carried out by researchers in our sample. Grit Laudel (2003) in her pioneer work of using bibliometrics to measure the magnitude of the brain drain recommended choosing a narrow subject of research, which in that case the research subject was ‘*angiotensin*’. The study of the elite scientists working on such a narrow subject provided a robust evidence of brain drain towards the U.S., rather than identifying those countries losing their elite scientists. However, a small country will have if any, an insignificant number of elite scientists in such a narrow field of research mainly because it might not be within traditional subjects and profiles of the country of origin, as it was shown in less favoured regions of the European Communities (Narin and Whitlow 1990). Further studies will focus instead, on leadership and excellence as bibliometric indicators and their relation to Cuban output in science (Arencibia-Jorge et al. 2016).

Our results support the view that mobile researchers and the destination countries are the obvious beneficiaries from the mobility and migration of scientists. The researchers themselves have increased their scientific productivity (from 552 to 1863 articles, Table 1) and have diversified their collaboration, becoming part of the global network of science and transnational researchers with strong ties to Latin American and the Caribbean region. Europe has acquired talented collaborative and productive researchers that support Europe’s ties to Latin American and Caribbean region. The contribution of Cuban researchers to the European output in science (*P*_E_) strongly depends on the capacity of these researchers to collaborate (*C*_E_), including their collaboration with the home country (*C*_C_). Scientific collaboration was found to be a strong predictor of publishing productivity especially during the period of academic career development (Lee and Bozeman 2005). Indeed, researchers themselves establish their network of collaborators within the field of research (Melin 2000) with strong influence of teamwork in earlier years of their careers. The finding that the collaboration with the home country (*C*_C_) strongly depends on the CRiE performance in Europe (*P*_E_ and *C*_E_) points to a positive side of the mobility/migration of scientists in relation to their country of origin.

This study indicates that Cuban scientific capacity supports a platform for individual scientific development of Cuban researchers in the country and abroad. This seems to be a direct consequence of the Cuban policy during and after the 90s in which post-graduate activities were extended beyond the borders (Nuñez Jover 2010, 129). Thus, some Cuban academics were taking contracts abroad becoming nodes of new networks of international collaboration as well as providing an important economic contribution to Cuban universities. The author also discussed how the implementation of new policies was having two opposite sides: inserting Cuban science in the international network of collaboration and, on the other side, some researchers were moving away from topics related to the need of the Cuban society, as they were pursuing to publish in *main stream* journals.

The above observation is in agreement with our findings, in which the collaboration of CRiE with Cuba decreases over time since the moment they start publishing with European affiliations. One explanation for this trend might be that Cuban researchers, once working in Europe need to adapt their interests to those in their new destinations, as they receive funding from European agencies, governments and sometimes, international sources. Those interests do not always coincide with previous priorities in the home country, and to guarantee their jobs as scientists there is no other option than to follow new demands. This reasoning also leads to the reality under which these researchers compete: either they publish or perish, regardless of what might be important to the home country. This crucial aspect of the international collaboration between developed and developing country required attention. Further studies should follow in order to identify how the ties of mobile Cuban researchers could be strengthening to ensure a sustainable benefit for the home country. The model and methodology used in this study did not help to identify the proportion of Cuban researchers abroad that established new channels of collaboration between Cuba and Europe, or those who used previously established channels drawing out researchers from Cuba to Europe. We found that different situations were present in a sample of ten CRiE interviewed, but in all cases they wanted first to progress in their careers having access to global connections and resources.

The academic sector dominates the network of collaboration of Cuban researchers in Europe, as all of them were associated to institutions of HE at a certain stage of their careers in countries of the region. Since the end of the Cold War, the European Union has consistently promoted international mobility of academic students through different programs of collaboration and exchanges including emerging regions such as Latin America, assisting in part the process of internationalization of higher education in this region. Reconfiguration of the international collaboration and the internationalization of HE in Cuba took place after the collapse of the socialist countries in Eastern Europe and the Soviet Union (Palacios-Callender et al. 2016). Cuba with more than 75% of cooperation and exchanges with the socialist countries had to adopt emerging measures to counteract the sudden collapse of those collaborations between 1989 and 1991. By 2001, Latin American and Caribbean countries were the main partners of the Cuban higher education system accounting for 72% of cooperation related activities, followed by Spain with 14% and the rest with the European Union, Canada and the United States. Between 1996 and 2002, the official number of Cuban undergraduate and graduate students granted scholarships to study abroad was 350 (Hernández Pérez 2005). Interestingly, although Spain has been the main destination country of Cuban researchers in Europe, the country seems to bridge the mobility of researchers to other destinations in Europe, mainly United Kingdom, which is a magnet to develop academic careers (Bhandari and Blumenthal 2011; Aceituno-Aceituno et al. 2013, 2017). This effect was probably exacerbated by the global economic crisis during the period of study.

The high representation of Latin American institutions in the CRiE network of collaboration might be a consequence of both the increasing collaboration of Latin American countries with Europe (Russell and Ainsworth 2013) and the integration of Cuba to the region within the process of internationalization of HE (Hernández Pérez 2005; Nuñez Jover 2010, 117). Although the CRiE sample does not fully represent all Cuban researchers in Europe, these one hundred and seven active researchers have contributed to the Cuban collaboration with Europe by 3.8% (203 out of 5374 Cuban articles^8^ in collaboration with Europe) with the potential to extend Cuban connectivity, in particular the academic sector, with top universities in Europe and the world. More than an overlapping of collaborating institutions with the traditional Cuban partners, the 115 institutions where CRiE have been working represent new *nodes* for extending the Cuban network of cooperation. There are institutions in the core six of the CRiE network which are not among the main collaborators of Cuba such as University of Oxford, University College London, Imperial College of Science Technology and Medicine in the United Kingdom, Karolinska Institute in Sweden, Spanish National Cancer Research, etc. Almost three quarters of CRiE output is in co-authorship with at least another institution (1348 out of 1863 articles) generating a network of collaboration with the potential not only to increase the Cuban international collaboration (37.8%) in number, but in terms of visibility and recognition of Cuban advances in science and innovation. The main set of journals chosen by CRiE and collaborators to publish their results is another potential source of diversity for the visibility of Cuba. It is reassuring to find that the top Cuban universities (University of Havana and Central University of Las Villas) contributing to 18% of national output in science with 4496 articles (see footnote 7) are in a central position of the CRiE network with other top universities worldwide. Another study using Science Citation Index as a bibliographic source found similar results (Herrera-Vallejera et al. 2017). The authors of this scientometric study of Cuban output between 2010 and 2012 showed the relevant positions of University of Havana and the Central University of Villa Clara and their central positions in the networks of collaboration in Chemistry, Physical Chemistry, Nanosciences/Nanotechnology and Engineering.

Some countries in Latin America experiencing lost of talents during economic crisis have implemented policies aiming to stop the flow of researchers going abroad. The inclusion of the Mexican scientific diaspora in the National System of Researchers (SNI) in 2009 allowed the evaluation of the impact that Mexican mobile researchers had to the Mexican international collaboration in science (Marmolejo-Leyva et al. 2015). Policies promoting the engagement of their mobile researchers abroad with national interests open new avenues to harness their potential created abroad. Moreover, such as in the case of SNI of Mexico, the implementation of the policy also created the opportunity to monitor the trends in order to improve the output in time. Nevertheless, science policy at the origin country also needs action from destination countries.

Foreign-born researchers in Europe from developing countries might effectively help their home countries through assisted transnational knowledge networks within current funding programs, in which all stakeholders are actively involved (Tejada 2012). Another successful international collaboration is VLIR-OUS^9^ programmes for inter-universities cooperation between universities and colleges in Flanders (Belgium) and developing countries. VLIR-OUS programmes ensure that junior researchers from developing countries who started their projects in top international institutions are able to continue their research back at home by providing complementary financial resources.

Science is a global enterprise accruing substantial contributions of all types of scientific collaboration measured as co-authorships of peer-reviewed publications in which the networks of international collaboration are a key feature of today’s generation of knowledge (The Royal Society 2011). The growth of such global networks is driven by a bottom-up self-organized collaboration of scientists participating in international projects challenging the way national systems operate (Wagner et al. 2015). The situation is even more challenging for small countries lacking the necessary resources to compete on the global stage and to attract top researchers to projects of national interest. The question arises how to measure and follow the scientific contribution of mobile researchers from small countries both globally and locally, in order to optimise benefits to all parts involved. Although emerging economies have been successful in designing and implementing policies to reverse their brain drain (Li 2004), ensuring their stake in the global networks of scientific collaboration (Jonkers and Tijssen 2008; Bornmann et al. 2015), the reality is that the size of the country matters. Small countries are more vulnerable to suffer from the mobility of scientists, ending in migration rather than ensuring through them, a place in science on the global stage because their pool of talents and resources are inevitably more limited.

## Conclusion

Mobility of students and academics is vital to any country interested in investing in science to promote development. However, an efficient system should be in place to harness the knowledge and skills emerging from the mobility of researchers working in global networks of science. National science systems should include policies and the mechanisms for harnessing the connectivity of their researchers abroad to ensure the country is dynamically embedded in the global network of international collaboration without losing the scope of local social needs.

Small developing countries are likely to find difficulties in sustaining the international collaboration with their academic researchers abroad. More research needs to be carried out to characterize this particular landscape. Governments of destination countries and international agencies can contribute to understanding this and address the sustainable collaboration in science with small countries in development.

Student mobility feeds the potential of the global network of knowledge as a public good. The trend to attract the best and brightest talents in the current environment of treating knowledge as a private good seems to be a threat to the universal right Louis Pasteur once advocated: ‘Knowledge belongs to humanity, and thus science knows no country and is the torch that illuminate the world’ (The Royal Society 2011, 14).

## Electronic supplementary material

Below is the link to the electronic supplementary material.

Appendix A: **CRiE 209-01** data downloaded from *Scopus* and the analysis for type of collaboration and nexus with Cuban institutions. Values of Pc, Pe, Ce, Cc are 0, 59, 57, 3 respectively. 209-01 published 1 and 2 articles after 5 and 8 years respectively of publishing in Europe. (DOCX 144 kb)

Appendix B: Aggregate values of P_C_, P_E_, C_E_, C_C_ of 107 CRiE according to their nexus with Cuba between 1995 and 2014. (DOCX 134 kb)

Appendix C: **CRiE 209-01** segment matrix. Using *Scopus* institutional addresses (sheet 1) a matrix (sheet 2) of three columns is created as Type I institution (CRiE affiliation), type II institution (collaborating) and number of their collaboration or links. The aggregate data of 107 CRiE is transformed in one symmetric matrix using ad-hoc program (Marcet García et al. 2016). (DOCX 105 kb)

Appendix D: **CRiE vs. CRiC**. Cuban researchers in Europe (106) were compared to top Cuban researchers (100) from 34 Cuban institutions in terms of their Seniority (S) and Productivity (P). (DOCX 356 kb)

## Footnotes

1 RICYT. Red Iberoamericana de indicadores de Ciencia y Técnica. *First level university graduates*. http://dev.ricyt.org/ui/v3/comparative.html?indicator=CGRADO&start_year=1990 [accessed: 7th February 2018].

2 CITMA (Ministerio de Ciencia Tecnologia y Medio Ambiente, Cuba) certified 195 scientific journals in 2016 http://www.citma.gob.cu/indicadores3/. By 2016, *Scopus* has included only 24 Cuban scientific journals in their database of 28,606 journals worldwide http://www.scimagojr.com/journalrank.php (accessed 07/04/2018).

3 Date of updating the search in *Scopus*: 17th April 2018.

4 This number refers to all Cuban-born scientists and engineers (s&e), including those not working in science and engineering (S&E) related occupations, those looking for jobs and those out of the labour force. The percentage of s&e directly involved in S&E occupations shown by the authors was 30.5%, but the Cuban s&e proportion was not specified (Kannankutty and Burrelli 2007).

5 In the following two independent cases, the collaboration was assigned to the Universities rather than CNRS (Le Centre National Recherche Scientifique) o CSIC (Consejo Superior de Investigaciones Científicas): *Université de Nice-Sophia Antipolis*, *Laboratoire de Chimie des Molécules Bioactives et des Aromes*, *UMR 6001 CNRS*, *Institut de Chimie de Nice*, *Faculté des Sciences*, *Parc Valrose*, *06108 Nice Cedex 02*, *France; Institut Català* *de Nano tecnologia* (*ICN*), *Centre d’Investigació en Nano ciència i Nano tecnologia* (*CSIC*), *Campus UAB* [*Universidad Autonoma de Barcelona*], *Edifice CM3*, *08193 Barcelona*, *Spain*.

6 In January 2013 restrictions to Cuban citizens to travel freely abroad was lifted, allowing the returning home within a period of 2 years (302 Act, 14-01-2013 modified the 1312 Act of Migration, 20-09-1976).

7 Search in Scopus using <affiliation country> engine: Cuba, limited to articles and reviews between 1995 and 2014. Subsequently using <analyze search> to find international institutions publishing with Cuba [accessed 07-04-2018].

8 Date of updating the search in *Scopus*: 17th April 2018.

9 VLIR-OUS: https://www.vliruos.be/en/home/1.
