# Supplementary material for: Scientific collaboration of Cuban researchers working in Europe: understanding relations between origin and destination countries
Source: Scientometrics. 2018 Aug 20;117(2):745–69. doi: 10.1007/s11192-018-2888-2 (PMC6280978; doi:10.1007/s11192-018-2888-2)
Supplement: Supplementary file 1 — CRiE 209-01 data downloaded from Scopus and the analysis for type of collaboration and nexus with Cuban institutions. Values of Pc, Pe, Ce, Cc are 0, 59, 57, 3 respectively. 209-01 published 1 and 2 articles after 5 and 8 years respectively of publishing in Europe. (DOCX 144 kb) [file 11192_2018_2888_MOESM1_ESM.docx]

APPENDIX A CRiE 209-01

Records of publications. Source: Scopus. Downloaded 2015/03/24

|  | **Scopus ID: 35723721100 CRiE code: 209-01** | **Title** | **Year** | **Source title** | **Affiliations/ article** | **Type of Collaboration** | **Cited by** | **Authors affiliations** |
| --- | --- | --- | --- | --- | --- | --- | --- | --- |
|  | Wydra K., Suder A., Borroto-Escuela D.O., Filip M., Fuxe K. | On the role of A2A and D2 receptors in control of cocaine and food-seeking behaviors in rats | 2014 | Psychopharmacology | 2 | NI |  | Laboratory of Drug Addiction Pharmacology, Department of Pharmacology, Institute of Pharmacology, Polish Academy of Sciences, Sm€ªtna 12KrakÌ_w, Poland; **Department of Neuroscience, Karolinska InstitutetStockholm, Sweden** |
|  | Borroto-Escuela D.O., Narvaez M., Perez-Alea M., Tarakanov A.O., Jimenez-Beristain A., Mudo G., Agnati L.F., Ciruela F., Belluardo N., Fuxe K. | Evidence for the existence of FGFR1-5-HT1A heteroreceptor complexes in the midbrain raphe 5-HT system | 2014 | Biochemical and Biophysical Research Communications | 5 | NI |  | **Department of Neuroscience, Karolinska Institutet, Stockholm, Sweden;** Department of Physiology, School of Medicine, University of MÌÁlaga, Spain; Lab Animal Models and Cancer Laboratory Anatomy Pathology Program, Institut de Recerca Vall d'Hebron, 08035 Barcelona, Spain; Russian Academy of Sciences, St. Petersburg Institute for Informatics and Automation, Saint Petersburg, Russia; Department of Experimental Biomedicine and Clinical Neurosciences, Laboratory of Molecular Neurobiology, University of Palermo, Palermo, Italy; Unitat de Farmacologia, Departament Patologia i TerapÌ¬utica Experimental, Universitat de Barcelona, Spain |
|  | Borroto-Escuela D.O., Narvaez M., Di Palma M., Calvo F., Rodriguez D., Millon C., Carlsson J., Agnati L.F., Garriga P., Diaz-Cabiale Z., Fuxe K. | Preferential activation by galanin 1-15 fragment of the GalR1 protomer of a GalR1-GalR2 heteroreceptor complex | 2014 | Biochemical and Biophysical Research Communications | 6 | NI |  | **Department of Neuroscience, Karolinska Institutet, Retzius vÌ_g 8Stockholm, Sweden**; Universidad de MÌÁlaga, Facultad de Medicina, Campus deTeatinos s/nMÌÁlaga, Spain; Department of Earth, Life and Environmental Sciences, Section of Physiology, Campus Scientifico Enrico MatteiUrbino, Italy; Department of Biochemistry and Biophysics, Stockholm UniversityStockholm, Sweden; Department of Biomedical Sciences, University of Modena, Italy; Departament d'Enginyeria QuÌ_mica, Universitat PolitÌ¬cnica de CatalunyaTerrassa, Spain |
|  | Borroto-Escuela D.O., Narvaez M., Di Palma M., Calvo F., Rodriguez D., Millon C., Carlsson J., Agnati L.F., Garriga P., Diaz-Cabiale Z., Fuxe K. | Preferential activation by galanin 1-15 fragment of the GalR1 protomer of a GalR1-GalR2 heteroreceptor complex | 2014 | Biochemical and Biophysical Research Communications | 6 | NNI |  | **Department of Neuroscience, Karolinska Institutet, Retzius vÌ_g 8, 17177 Stockholm, Sweden**; Universidad de MÌÁlaga, AndalucÌ_a Tech, Facultad de Medicina, Campus deTeatinos s/n, 29071 MÌÁlaga, Spain; Department of Earth, Life and Environmental Sciences, Section of Physiology, Campus Scientifico Enrico Mattei, Urbino, Italy; Department of Biochemistry and Biophysics, Stockholm University, Stockholm, Sweden; Department of Biomedical Sciences, University of Modena, Italy; Departament d'Enginyeria QuÌ_mica, Universitat PolitÌ¬cnica de Catalunya, Terrassa 08222, Spain |
|  | Romero-Fernandez W., Borroto-Escuela D.O., Vargas-Barroso V., Narvaez M., Di Palma M., Agnati L.F., Larriva Sahd J., Fuxe K. | Dopamine D1 and D2 receptor immunoreactivities in the arcuate-median eminence complex and their link to the tubero-infundibular dopamine neurons | 2014 | European Journal of Histochemistry | 5 | NI |  | **Department of Neuroscience, Karolinska Institute, Stockholm, Sweden**; Technical University of Ambato, Ecuador; Institute of Neurobiology, National Autonomous University of Mexico, Campus Juriquilla, Mexico; University of Malaga, Andalucia Tech, Spain; Department of Earth, Life and Environmental Sciences, Section of Physiology, Carlo Bo University of Urbino, Italy |
|  | Narvaez M., Millon C., Borroto-Escuela D., Flores-Burgess A., Santin L., Parrado C., Gago B., Puigcerver A., Fuxe K., Narvaez J.A., Diaz-Cabiale Z. | Galanin receptor 2-neuropeptide Y Y1 receptor interactions in the amygdala lead to increased anxiolytic actions | 2014 | Brain Structure and Function | 2 | NI |  | Universidad de MÌÁlaga, Instituto de InvestigaciÌ_n BiomÌ©dica, Facultad de Medicina, MÌÁlaga, Spain; **Department of Neuroscience, Karolinska Institute, Stockholm, Sweden;** Universidad de MÌÁlaga, Instituto de InvestigaciÌ_n BiomÌ©dica, Facultad de Psicologia, MÌÁlaga, Spain; Universidad de MÌÁlaga, Instituto de InvestigaciÌ_n BiomÌ©dica, Facultad de Ciencias, MÌÁlaga, Spain |
|  | Borroto-Escuela D.O., Brito I., Romero-Fernandez W., Di Palma M., Oflijan J., Skieterska K., Duchou J., Van Craenenbroeck K., Suarez-Boomgaard D., Rivera A., Guidolin D., Agnati L.F., Fuxe K. | The G protein-coupled receptor heterodimer network (GPCR-HetNet) and its hub components | 2014 | International Journal of Molecular Sciences | 7 | NI | 2 | **Department of Neuroscience, Karolinska Institutet, Retzius vÌ_g 8, 17177 Stockholm, Sweden**; IIIA-CSIC, Artificial Intelligence Research Institute, Spanish National Research Council, 08193 Barcelona, Spain; Department of Earth,Life and Environmental Sciences, Section of Physiology, Campus Scientifico Enrico Mattei, Urbino 61029, Italy; Department of Physiology, University of Tartu, Tartu 50411, Estonia; Laboratory of Eukaryotic Gene Expression and Signal Transduction (LEGEST), Ghent University, 9000 Ghent, Belgium; Department of Cell Biology, School of Science, University of MÌÁlaga, 29071 MÌÁlaga, Spain; Department of Molecular Medicine, University of Padova, Padova 35121, Italy |
|  | Suarez-Boomgaard D., Gago B., Valderrama-Carvajal A., Roales-Bujan R., Van Craenenbroeck K., Duchou J., Borroto-Escuela D.O., Medina-Luque J., de la Calle A., Fuxe K., Rivera A. | Dopamine D4 receptor counteracts morphine-induced changes in ë_ opioid receptor signaling in the striosomes of the rat caudate putamen | 2014 | International Journal of Molecular Sciences | 5 | NI |  | Department of Cell Biology, School of Science, University of MÌÁlaga, 29071 MÌÁlaga, Spain; Department of Neuroscience, Biodonostia Institute, 20014 San SebastiÌÁn, Spain; Laboratory of Eukaryotic Gene Expression and Signal Transduction (LEGEST), Ghent University-Gent, 9000 Ghent, Belgium; **Department of Neuroscience, Karolinska Institutet, Retzius vÌ_g 8, 17177 Stockholm, Sweden** |
|  | Borroto-Escuela D.O., Romero-Fernandez W., Narvaez M., Oflijan J., Agnati L.F., Fuxe K. | Hallucinogenic 5-HT2AR agonists LSD and DOI enhance dopamine D2R protomer recognition and signaling of D2-5-HT2A heteroreceptor complexes | 2014 | Biochemical and Biophysical Research Communications | 3 | NI | 5 | **Department of Neuroscience, Karolinska Institutet, Stockholm, Sweden**; Department of Physiology, School of Medicine, University of MÌÁlaga, Spain; Department of Physiology, Faculty of Medicine, University of Tartu, Estonia; IRCCS Lido, Venice, Italy |
|  | Navarro G., Borroto-Escuela D.O., Fuxe K., Franco R. | Potential of caveolae in the therapy of cardiovascular and neurological diseases | 2014 | Frontiers in Physiology | 2 | NI |  | Departamentde Bioqui'mica i Biologia Molecular, Facultatde Biologia, Universitat de BarcelonaBarcelona, Spain**; Department of Neuroscience, Karolinska InstitutetStockholm, Sweden** |
|  | Fuxe K., Agnati L.F., Borroto-Escuela D.O. | The impact of receptor-receptor interactions in heteroreceptor complexes on brain plasticity | 2014 | Expert Review of Neurotherapeutics | 2 | NI |  | **Department of Neuroscience, Division of Cellular and Molecular Neurochemistry, Karolinska Institutet, 17177 Stockholm, Sweden;** IRCCS San Camillo, Via Alberoni 70, 30126-Lido, Venezia, Italy |
|  | Fuxe K., Borroto-Escuela D.O., Romero-Fernandez W., Palkovits M., Tarakanov A.O., Ciruela F., Agnati L.F. | Moonlighting proteins and protein-protein interactions as neurotherapeutic targets in the G protein-coupled receptor field | 2014 | Neuropsychopharmacology | 4 | NI | 5 | **Department of Neuroscience, Karolinska Institutet, Stockholm, Sweden;** Department of Anatomy, Histology and Embryology, Hungarian Academy of Sciences and Semmelweis University, Budapest, Hungary; Russian Academy of Sciences, St. Petersburg Institute for Informatics and Automation, St-Petersburg, Russian Federation; Facultat de Medicina, Departament de Patologia i TerapÌ¬utica Experimental IDIBELL-Universitat de Barcelona, L'Hospitalet de Llobregat, Unitat de Farmacologia, Barcelona, Spain; IRCCS, Lido Venice, Italy |
|  | Fuxe K., Borroto-Escuela D.O., Tarakanov A.O., Romero-Fernandez W., Ferraro L., Tanganelli S., Perez-Alea M., Di Palma M., Agnati L.F. | Dopamine D2 heteroreceptor complexes and their receptor-receptor interactions in ventral striatum: Novel targets for antipsychotic drugs | 2014 | Progress in Brain Research | 6 | NI | 1 | **Department of Neuroscience, Karolinska Institutet, Stockholm, Sweden;** Russian Academy of Sciences, St Petersburg Institute for Informatics and Automatation, St. Petersburg, Russian Federation; Department of Life Sciences and Biotechnology, University of Ferrara, Ferrara, Italy; Department of Pathology, Hospital Universitari Vall d'Hebron, Barcelona, Spain; Department of Earth, Life and Environmental Sciences, Section of Physiology, Campus Scientifico 'Enrico Mattei', Urbino, Italy; IRCCS San Camillo, Lido Venice, Italy |
|  | Agnati L.F., Guidolin D., Maura G., Marcoli M., Leo G., Carone C., De Caro R., Genedani S., Borroto-Escuela D.O., Fuxe K. | Information handling by the brain: proposal of a new ‰ÛÏparadigm‰Û involving the roamer type of volume transmission and the tunneling nanotube type of wiring transmission | 2014 | Journal of Neural Transmission | 4 | NI |  | Department of Biomedical Sciences, University of Modena and Reggio Emilia, Via Campi, 287Modena, Italy; Department of Molecular Medicine, University of Padova, Via Gabelli, 65Padua, Italy; Department of Pharmacy and Center of Excellence for Biomedical Research (CEBR), University of GenovaGenoa, Italy; **Department of Neuroscience, Karolinska InstitutetStockholm, Sweden** |
|  | Fuxe K., Tarakanov A., Romero Fernandez W., Ferraro L., Tanganelli S., Filip M., Agnati L.F., Garriga P., Diaz-Cabiale Z., Borroto-Escuela D.O. | Diversity and bias through receptor-receptor interactions in GPCR heteroreceptor complexes. Focus on examples from dopamine D2 receptor heteromerization | 2014 | Frontiers in Endocrinology | 7 | NI |  | **Department of Neuroscience, Karolinska Institutet, Stockholm, Sweden;** St. Petersburg Institute for Informatics and Automation, Russian Academy of Sciences, Saint Petersburg, Russian Federation; Pharmacology Section, Department of Clinical and Experimental Medicine, University of Ferrara, Ferrara, Italy; Laboratory of Drug Addiction Pharmacology, Department of Pharmacology, Institute of Pharmacology, Polish Academy of Sciences, KrakÌ_w, Poland; Istituto di Ricovero e Cura a Carattere Scientifico, Venice Lido, Italy; Departament d'Enginyeria QuÌ_mica, Universitat PolitÌ¬cnica de Catalunya, Barcelona, Spain; Department of Physiology, School of Medicine, University of MÌÁlaga, MÌÁlaga, Spain |
|  | Fuxe K., Borroto-Escuela D.O., Tarakanov A., Fernandez W.R., Manger P., Rivera A., Van Craenenbroeck K., Skieterska K., Diaz-Cabiale Z., Filip M., Ferraro L., Tanganelli S., Guidolin D., Cullheim S., De La Mora M.P., Agnati L.F. | Understanding the balance and integration of volume and synaptic transmission. Relevance for psychiatry | 2013 | Neurology Psychiatry and Brain Research | 11 | NI | 2 | **Department of Neuroscience, Karolinska Institute, Stockholm, Sweden;** Russian Academy of Sciences, St. Petersburg Institute for Informatics and Automation, Saint Petersburg, Russian Federation; School of Anatomical Sciences, Faculty of Health Sciences, University of the Witwatersrand, Johannesburg, South Africa; Department of Cell Biology, Faculty of Sciences, University of MÌÁlaga, Spain; Laboratory of Eukaryotic Gene Expression and Signal Transduction (LEGEST), Ghent University-Gent, Belgium; Department of Physiology, School of Medicine, University of MÌÁlaga, Spain; Department of Pharmacology, Institute of Pharmacology, Polish Academy of Sciences, Smetna 12, PL 31-343 KrakÌ_w, Poland; Department of Clinical and Experimental Medicine, Pharmacology Section, University of Ferrara, Italy; Department of Human Anatomy and Physiology, University of Padova, Italy; Department of Biophysic, Instituto de FisiologÌ_a Celular, Universidad Nacional AutÌ_noma de MÌ©xico, MÌ©xico, DF, Mexico; IRCCS Lido Venice, Italy; Department of Toxicology, Faculty of Pharmacy, Jagiellonian University, KrakÌ_w, Poland |
|  | Borroto-Escuela D.O., Corrales F., Narvaez M., Oflijan J., Agnati L.F., Palkovits M., Fuxe K. | Dynamic modulation of FGFR1-5-HT1A heteroreceptor complexes. Agonist treatment enhances participation of FGFR1 and 5-HT1A homodimers and recruitment of ë_-arrestin2 | 2013 | Biochemical and Biophysical Research Communications | 6 | NI | 2 | **Department of Neuroscience, Karolinska Institutet, Stockholm, Sweden;** Centro Nacional de Neurociencias, La Habana, Cuba; Department of Physiology, School of Medicine, University of MÌÁlaga, Spain; Department of Physiology, Faculty of Medicine, University of Tartu, Estonia; IRCCS Lido Venice, Italy; Magyar Tudomanyos Akademia-Semmelweis Egyetem Neuromorfologiai Es Neuroendokrin Kutatocsoport, TuzoltÌ_ u. 58, Budapest 1094, Hungary |
|  | Borroto-Escuela D.O., Flajolet M., Agnati L.F., Greengard P., Fuxe K. | Bioluminescence Resonance Energy Transfer Methods to Study G Protein-Coupled Receptor-Receptor Tyrosine Kinase Heteroreceptor Complexes | 2013 | Methods in Cell Biology | 3 | NI | 5 | **Department of Neuroscience, Karolinska Institutet, Stockholm, Sweden;** Laboratory of Molecular and Cellular Neuroscience, The Rockefeller University, New York, United States; IRCCS Lido, Venice, Italy |
|  | Borroto-Escuela D.O., Romero-Fernandez W., Rivera A., Van Craenenbroeck K., Tarakanov A.O., Agnati L.F., Fuxe K. | On the G-protein-coupled receptor heteromers and their allosteric receptor-receptor interactions in the central nervous system: Focus on their role in pain modulation | 2013 | Evidence-based Complementary and Alternative Medicine | 6 | NI | 4 | **Department of Neuroscience, Karolinska Institutet, Retzius vÌ_g 8, 17177 Stockholm, Sweden;** Faculty of Science, University of Malaga, 29080 Malaga, Spain; Laboratory of Eukaryotic Gene Expression and Signal Transduction (LEGEST), Ghent University-Gent, 9000 Ghent, Belgium; Russian Academy of Sciences, St. Petersburg Institute for Informatics and Automation, 193167 Saint-Petersburg, Russian Federation; IRCCS, Lido Venice, 41100 Venice, Italy |
|  | Borroto-Escuela D.O., Romero-Fernandez W., Rivera A., Van Craenenbroeck K., Tarakanov A.O., Agnati L.F., Fuxe K. | On the G-protein-coupled receptor heteromers and their allosteric receptor-receptor interactions in the central nervous system: Focus on their role in pain modulation | 2013 | Evidence-based Complementary and Alternative Medicine | 6 | NI | 4 | **Department of Neuroscience, Karolinska Institutet, Retzius vÌ_g 8, 17177 Stockholm, Sweden;** Faculty of Science, University of Malaga, 29080 Malaga, Spain; Laboratory of Eukaryotic Gene Expression and Signal Transduction (LEGEST), Ghent University-Gent, 9000 Ghent, Belgium; Russian Academy of Sciences, St. Petersburg Institute for Informatics and Automation, 193167 Saint-Petersburg, Russian Federation; IRCCS, Lido Venice, 41100 Venice, Italy |
|  | Romero-Fernandez W., Borroto-Escuela D.O., Agnati L.F., Fuxe K. | Evidence for the existence of dopamine d2-oxytocin receptor heteromers in the ventral and dorsal striatum with facilitatory receptor-receptor interactions | 2013 | Molecular Psychiatry | 1 | N | 14 | **Department of Neuroscience, Karolinska Institutet, Stockholm, Sweden** |
|  | Borroto-Escuela D.O., Ravani A., Tarakanov A.O., Brito I., Narvaez M., Romero-Fernandez W., Corrales F., Agnati L.F., Tanganelli S., Ferraro L., Fuxe K. | Dopamine D2 receptor signaling dynamics of dopamine D2-neurotensin 1 receptor heteromers | 2013 | Biochemical and Biophysical Research Communications | 7 | NI | 6 | **Department of Neuroscience, Karolinska Institutet, Stockholm, Sweden;** Department of Clinical and Experimental Medicine, Pharmacology Section and LTTA Centre, University of Ferrara, Ferrara, Italy; Russian Academy of Sciences, St. Petersburg Institute for Informatics and Automation, Saint Petersburg, Russian Federation; III-A, CSIC, Barcelona, Spain; Department of Physiology, School of Medicine, University of MÌÁlaga, Spain; Centro Nacional de Neurociencias, La Habana, Cuba; IRCCS Lido Venice, Italy |
|  | Fuxe K., Borroto-Escuela D.O., Romero-Fernandez W., Zhang W.-B., Agnati L.F. | Volume transmission and its different forms in the central nervous system | 2013 | Chinese Journal of Integrative Medicine | 3 | NI | 10 | **Department of Neuroscience, Karolinska Institutet, Stockholm, Sweden;** Institute of Acupuncture and Moxibustion, China Academy of Chinese Medical Sciences, Beijing 100700, China; Department of Biomedical Sciences, University of Modena, Modena, Italy |
|  | Borroto-Escuela D.O., Romero-Fernandez W., Garriga P., Ciruela F., Narvaez M., Tarakanov A.O., Palkovits M., Agnati L.F., Fuxe K. | G protein-coupled receptor heterodimerization in the Brain | 2013 | Methods in Enzymology | 7 | NI | 15 | **Department of Neuroscience, Karolinska Institutet, Stockholm, Sweden;** Departament d'Enginyeria QuÌ_mica, Universitat PolitÌ¬cnica de Catalunya, Barcelona, Spain; Unitat de Farmacologia, Departament Patologia i TerapÌ¬utica Experimental, Universitat de Barcelona, Barcelona, Spain; Department of Physiology, School of Medicine, University of MÌÁlaga, MÌÁlaga, Spain; Russian Academy of Sciences, St. Petersburg Institute for Informatics and Automation, Saint-Petersburg, Russian Federation; Human Brain Tissue Bank, Semmelweis University, Budapest, Hungary; IRCCS Lido, Venice, Italy |
|  | Fuxe K., Borroto-Escuela D.O., Romero-Fernandez W., Tarakanov A.O., Calvo F., Garriga P., Tena M., Narvaez M., Millon C., Parrado C., Ciruela F., Agnati L.F., Narvaez J.A., Diaz-Cabiale Z. | On the existence and function of galanin receptor heteromers in the central nervous system | 2012 | Frontiers in Endocrinology | 6 | Ni | 5 | **Department of Neuroscience, Karolinska Institutet, Stockholm, Sweden;** St. Petersburg Institute for Informatics and Automation, Russian Academy of Sciences, Saint Petersburg, Russian Federation; Centre de Biotecnologia Molecular, Departament díEnginyeria QuÌ_mica, Universitat PolitÌ©cnica de Catalunya, Barcelona, Spain; Department of Physiology, School of Medicine, University of MÌÁlaga, MÌÁlaga, Spain; Department of Histology, School of Medicine, University of MÌÁlaga, MÌÁlaga, Spain; Unitat de Farmacologia, Departament Patologia iTerapÌ©utica Experimental, Universitat de Barcelona, Barcelona, Spain; Department of Biomedical Sciences, University of Modena and Reggio Emilia, Modena, Italy; Istituto di Ricovero e Cura a Carattere Scientifico, LidoVenice, Italy |
|  | Fernandez-Duenas V., Gomez-Soler M., Jacobson K.A., Kumar S.T., Fuxe K., Borroto-Escuela D.O., Ciruela F. | Molecular determinants of A 2AR-D 2R allosterism: Role of the intracellular loop 3 of the D 2R | 2012 | Journal of Neurochemistry | 3 | NI | 8 | Unitat de Farmacologia, Facultat de Medicina, L'Hospitalet de Llobregat, Barcelona 08907, Spain; Laboratory of Bioorganic Chemistry, National Institute of Diabetes and Digestive and Kidney Diseases, National Institutes of Health, Bethesda, MD, United States; **Department of Neuroscience, Karolinska Institutet, Stockholm, Sweden** |
|  | Agnati L.F., Barlow P., Ghidoni R., Borroto-Escuela D.O., Guidolin D., Fuxe K. | Possible genetic and epigenetic links between human inner speech, schizophrenia and altruism | 2012 | Brain Research | 5 | NI | 6 | IRCCS San Camillo, via Alberoni 70, 30126 Venezia Lido (VE), Italy; School of Biological Sciences, University of Bristol, Bristol, United Kingdom; Proteomics Unit, IRCCS Centro S. Giovanni di Dio-Fatebenefratelli, Brescia, Italy; **Department of Neuroscience, Karolinska Institutet, Stockholm, Sweden;** Department of Molecular Medicine, University of Padova, Padova, Italy |
|  | Fuxe K., Borroto-Escuela D.O., Romero-Fernandez W., Ciruela F., Manger P., Leo G., Diaz-Cabiale Z., Agnati L.F. | On the role of volume transmission and receptor-receptor interactions in social behaviour: Focus on central catecholamine and oxytocin neurons | 2012 | Brain Research | 6 | NI | 15 | **Department of Neuroscience, Karolinska Institutet, Stockholm, Sweden;** Unitat de Farmacologia, Departament Patologia i TerapÌ¬utica Experimental, Universitat de Barcelona, Spain; School of Anatomical Sciences, Faculty of Health Sciences, University of the Witwatersrand, 7 York Road, Parktown, 2193, South Africa; Department of BioMedical Sciences, University of Modena and Reggio Emilia, 41100 Modena, Italy; Department of Physiology, School of Medicine, University of MÌÁlaga, Spain; IRCCS Lido Venice, Italy |
|  | Ciruela F., Fernandez-Duenas V., Llorente J., Borroto-Escuela D., Cuffi M.L., Carbonell L., Sanchez S., Agnati L.F., Fuxe K., Tasca C.I. | G protein-coupled receptor oligomerization and brain integration: Focus on adenosinergic transmission | 2012 | Brain Research | 4 | NI | 5 | Unitat de Farmacologia, Departament de Patologia i TerapÌ¬utica Experimental, Hospitalet Del Llobregat, Av. Feixa Llarga, s/n, 08907, Barcelona, Spain**; Department of Neuroscience, Karolinska Institutet, Stockholm, Sweden;** IRCCS San Camillo Lido, 30100 Venezia, Italy; Departamento de BioquÌ_mica, Centro de CiÌ»ncias BiolÌ_gicas, Universidade Federal de Santa Catarina, Trindade, 88040-900 FlorianÌ_polis, SC, Brazil |
|  | Fuxe K., Borroto-Escuela D.O., Romero-Fernandez W., Diaz-Cabiale Z., Rivera A., Ferraro L., Tanganelli S., Tarakanov A.O., Garriga P., Narvaez J.A., Ciruela F., Guescini M., Agnati L.F. | Extrasynaptic neurotransmission in the modulation of brain function. Focus on the striatal neuronal-glial networks | 2012 | Frontiers in Physiology | 8 | NI | 18 | **Department of Neuroscience, Karolinska Institutet, Stockholm, Sweden;** Department of Physiology, School of Medicine, University of Malaga, Malaga, Spain; Department of Cell Biology, Faculty of Sciences, University of Malaga, Malaga, Spain; Pharmacology Section, Department of Clinical and Experimental Medicine, University of Ferrara, Ferrara, Italy; Russian Academy of Sciences, St. Petersburg Institute for Informatics and Automation, Saint Petersburg, Russian Federation; Departament d'Enginyeria Quimica, Centre de Biotecnologia Molecular, Universitat Politecnica de Catalunya, Barcelona, Spain; Unitat de Farmacologia, Departament Patologia i Terapeutica Experimental, Universitat de Barcelona, Barcelona, Spain; Department of Biomolecular Sciences, University of Urbino CarloBo, Urbino, Italy; IRCCS Lido, Venice, Italy |
|  | Tarakanov A.O., Fuxe K.G., Borroto-Escuela D.O. | Integrin triplets of marine sponges in human brain receptor heteromers | 2012 | Journal of Molecular Neuroscience | 2 | NI | 5 | Russian Academy of Sciences, St. Petersburg Institute for Informatics and Automation, Saint Petersburg, Russian Federation; **Department of Neuroscience, Karolinska Institute, Stockholm, Sweden** |
|  | Tarakanov A.O., Fuxe K.G., Borroto-Escuela D.O. | Integrin triplets of marine sponges in human D2 receptor heteromers | 2012 | Journal of Receptors and Signal Transduction | 2 | NI | 7 | Russian Academy of Sciences, St. Petersburg Institute for Informatics and Automation, Saint Petersburg, Russian Federation; **Department of Neuroscience, Karolinska Institutet, Stockholm, Sweden** |
|  | Fernandez-Duenas V., Llorente J., Gandia J., Borroto-Escuela D.O., Agnati L.F., Tasca C.I., Fuxe K., Ciruela F. | Fluorescence resonance energy transfer-based technologies in the study of protein-protein interactions at the cell surface | 2012 | Methods | 4 | NI | 10 | Unitat de Farmacologia, Departament de Patologia i TerapÌ¬utica Experimental, Facultat de Medicina, Universitat de Barcelona, L'Hospitalet de Llobregat, 08907 Barcelona, Spain**; Department of Neuroscience, Karolinska Institutet, Stockholm, Sweden;** IRCCS San Camillo Lido, 30100 Venezia, Italy; Departamento de BioquÌ_mica Centro de CiÌ»ncias BiolÌ_gicas, Universidade Federal de Santa Catarina, Florianopolis, SC 88040-900, Brazil |
|  | Tarakanov A.O., Fuxe K.G., Borroto-Escuela D.O. | On the origin of the triplet puzzle of homologies in receptor heteromers: Toll-like receptor triplets in different types of receptors | 2012 | Journal of Neural Transmission | 2 | NI | 6 | St. Petersburg Institute for Informatics and Automation, Russian Academy of Sciences, Saint Petersburg, Russian Federation; **Department of Neuroscience, Karolinska Institute, Stockholm, Sweden** |
|  | Guescini M., Leo G., Genedani S., Carone C., Pederzoli F., Ciruela F., Guidolin D., Stocchi V., Mantuano M., Borroto-Escuela D.O., Fuxe K., Agnati L.F. | Microvesicle and tunneling nanotube mediated intercellular transfer of g-protein coupled receptors in cell cultures | 2012 | Experimental Cell Research | 6 | NI | 13 | Department of Biomolecular Sciences, University of Urbino Carlo Bo, 61029 Urbino, Italy; Department Biomedical Sciences, University of Modena and Reggio Emilia, Italy; Departament Patologia i TerapÌ¬utica Experimental, Universitat de Barcelona, Spain; Department of Human Anatomy and Physiology, University of Padua, Italy; **Department of Neuroscience, Karolinska Institutet, Stockholm, Sweden;** IRCCS San Camillo Lido, Venezia, Italy |
|  | Tarakanov A.O., Fuxe K.G., Borroto-Escuela D.O. | On the origin of the triplet puzzle of homologies in receptor heteromers: Immunoglobulin triplets in different types of receptors | 2012 | Journal of Molecular Neuroscience | 2 | NI | 6 | St. Petersburg Institute for Informatics and Automation, Russian Academy of Sciences, Saint Petersburg, Russian Federation; **Department of Neuroscience, Karolinska Institute, Stockholm, Sweden** |
|  | Borroto-Escuela D.O., Romero-Fernandez W., Mudo G., Perez-Alea M., Ciruela F., Tarakanov A.O., Narvaez M., Di Liberto V., Agnati L.F., Belluardo N., Fuxe K. | Fibroblast growth factor receptor 1 5-hydroxytryptamine 1A heteroreceptor complexes and their enhancement of hippocampal plasticity | 2012 | Biological Psychiatry | 7 | NI | 19 | **Department of Neuroscience, Karolinska Institutet, Retzius vÌ_g 8, Stockholm 17177, Sweden;** Department of Experimental Biomedicine and Clinical Neurosciences, Laboratory of Molecular Neurobiology, University of Palermo, Palermo, Italy; School of Life and Health, Aston University, Birmingham, United Kingdom; Unitat de Farmacologia, Departament Patologia i Teraputica Experimental, Universitat de Barcelona, Spain; Russian Academy of Sciences, St. Petersburg Institute for Informatics and Automation, Saint Petersburg, Russian Federation; Department of Physiology, School of Medicine, University of MÌÁlaga, MÌÁlaga, Spain; IRCCS Ospedale San Camillo Lido, Venice, Italy |
|  | Fuxe K., Borroto-Escuela D.O., Marcellino D., Romero-Fernandez W., Frankowska M., Guidolin D., Filip M., Ferraro L., Woods A.S., Tarakanov A., Ciruela F., Agnati L.F., Tanganelli S. | GPCR heteromers and their allosteric receptor-receptor interactions | 2012 | Current Medicinal Chemistry | 8 | NI | 29 | **Department of Neuroscience, Karolinska Institutet, RetziusvÌ_g 8, 17177 Stockholm, Sweden;** Laboratory of Drug Addiction Pharmacology, Department of Pharmacology, Institute of Pharmacology, Smetna 12, PL 31-343 KrakÌ_w, Poland; Department of Human Anatomy and Physiology, University of Padova, Italy; Department of Clinical and Experimental Medicine, Pharmacology Section and LTTA Centre, University of Ferrara, Ferrara, Italy; NIDA-IRP, Structural Biology Unit, United States; Russian Academy of Sciences, St. Petersburg Institute for Informatics and Automation, St. Petersburg, Russian Federation; Unitat de Farmacologia, Facultat de Medicina, Universitat de Barcelona, Barcelona, Spain; IRCCS, Ospedali San Camillo, Venice, Italy |
|  | Borroto-Escuela D.O., Agnati L.F., Fuxe K., Ciruela F. | Muscarinic acetylcholine receptor-interacting proteins (mAChRIPs): Targeting the receptorsome | 2012 | Current Drug Targets | 3 | NI | 4 | **Department of Neuroscience, Karolinska Institutet, Retzius vÌ_g 8, 17177 Stockholm, Sweden;** IRCCS San Camillo, Lido Venezia, Italy; Unitat de Farmacologia, Departament Patologia i TerapÌ¬utica Experimental, Facultat de Medicina-Bellvitge, Universitat de Barcelona, PavellÌ_ de Govern, Av. Feixa Llarga, s/n, L'Hospitalet de Llobregat, 08907 Barcelona, Spain |
|  | Borroto-Escuela D.O., Romero-Fernandez W., Garcia-Negredo G., Correia P.A., Garriga P., Fuxe K., Ciruela F. | Dissecting the conserved NPxxY motif of the M 3 muscarinic acetylcholine receptor: Critical role of Asp-7.49 for receptor signaling and multiprotein complex formation | 2011 | Cellular Physiology and Biochemistry | 4 | NI | 5 | Centre de Biotecnologia Molecular, Department d'Enginyeria QuÌ©mica, Universitat PolitÌ¬cnica de Catalunya, Terrassa, Spain; **Neuroscience Department, Karolinska Institutet, Stockholm, Sweden;** Unitat de Farmacologia, Departament de Patologia i TerapÌ¬utica Experimental, L'Hospitalet del Llobregat Barcelona, PavellÌ_ de Govern Av. Feixa Llarga s/n 08907, Spain; W.M. Keck Centre for Integrative Neuroscience, Department of Physiology, University of California, San Francisco, United States |
|  | Romero-Fernandez W., Borroto-Escuela D.O., Perez Alea M., Garcia-Mesa Y., Garriga P. | Altered trafficking and unfolded protein response induction as a result of M 3 muscarinic receptor impaired N-glycosylation | 2011 | Glycobiology | 4 | NI | 3 | Department d'Enginyeria QuÌ_mica, Centre de Biotecnologia Molecular, Universitat PolitÌ¬cnica de Catalunya, Edifici Gaia, Rbla San Nebridi s/n, Terrassa, 08222 Barcelona, Spain**; Department of Neuroscience, Karolinska Institutet, Stockholm, Sweden;** School of Life and Health, Aston University, Birmingham, United Kingdom; Institut d'Investigacions BiomÌ¬diques de Barcelona, Barcelona, Spain |
|  | Alea M.P., Borroto-Escuela D.O., Romero-Fernandez W., Fuxe K., Garriga P. | Differential expression of muscarinic acetylcholine receptor subtypes in Jurkat cells and their signaling | 2011 | Journal of Neuroimmunology | 3 | NI | 4 | Centre for Molecular Biotechnology, Department of Chemical Engineering, Technical University of Catalonia, Barcelona, Spain; School of Life and Health Sciences, Aston Triangle, Aston University, Birmingham, United Kingdom**; Department of Neuroscience, Karolinska Institutet, Retzius vÌ_g 8, 17177 Stockholm, Sweden** |
|  | Borroto-Escuela D.O., Tarakanov A.O., Guidolin D., Ciruela F., Agnati L.F., Fuxe K. | Moonlighting characteristics of G protein-coupled receptors: Focus on receptor heteromers and relevance for neurodegeneration | 2011 | IUBMB Life | 5 | NI | 22 | **Department of Neuroscience, Karolinska Institutet, Stockholm, Sweden;** Russian Academy of Sciences, St. Petersburg Institute for Informatics and Automation, St. Petersburg, Russian Federation; Department of Human Anatomy and Physiology, University of Padova, Padova, Italy; Unitat de Farmacologia, Facultat de Medicina, Universitat de Barcelona, Barcelona, Spain; IRCCS, Ospedali San Camillo, Venice, Italy; RetziusvÌ_g 8, 17177 Stockholm, Sweden |
|  | Romero-Fernandez W., Borroto-Escuela D.O., Tarakanov A.O., Mudo G., Narvaez M., Perez-Alea M., Agnati L.F., Ciruela F., Belluardo N., Fuxe K. | Agonist-induced formation of FGFR1 homodimers and signaling differ among members of the FGF family | 2011 | Biochemical and Biophysical Research Communications | 6 | NI | 11 | **Department of Neuroscience, Karolinska Institutet, Stockholm, Sweden;** Russian Academy of Sciences, St. Petersburg Institute for Informatics and Automation, Saint Petersburg, Russian Federation; Department of Experimental Biomedicine and Clinical Neurosciences, Division of Human Physiology, Laboratory of Molecular Neurobiology, University of Palermo, Corso Tukory 129, 90134 Palermo, Italy; Department of Physiology, School of Medicine, University of MÌÁlaga, Spain; School of Life and Health, Aston University, Birmingham, United Kingdom; IRCCS, Lido Venice, Italy; Unitat de Farmacologia, Departament Patologia i TerapÌ¬utica Experimental, Universitat de Barcelona, Spain |
|  | Ciruela F., Gomez-Soler M., Guidolin D., Borroto-Escuela D.O., Agnati L.F., Fuxe K., Fernandez-Duenas V. | Adenosine receptor containing oligomers: Their role in the control of dopamine and glutamate neurotransmission in the brain | 2011 | Biochimica et Biophysica Acta - Biomembranes | 4 | NI | 23 | Unitat de Farmacologia, Dept. de Patologia i TerapÌ¬utica Experimental, Facultat de Medicina-Bellvitge, Av. Feixa Llarga, s/n, 08907 L'Hospitalet del Llobregat, Barcelona, Spain; Department of Human Anatomy and Physiology, University of Padova, Padova, Italy; **Department of Neuroscience, Karolinska Institutet, Stockholm, Sweden;** IRCCS San Camillo, Lido Venezia, Italy |
|  | Romero-Fernandez W., Garriga P., Borroto-Escuela D.O. | Overproduction of human M3 muscarinic acetylcholine receptor: An approach toward structural studies | 2011 | Biotechnology Progress | 2 | NI | 1 | Centre de Biotecnologia Molecular, Dept. d'Enginyeria QuÌ_mica, Universitat PolitÌ¬cnica de Catalunya, Terrassa 08222, Spain; **Dept. of Neuroscience, Karolinska Institutet, Stockholm 17177, Sweden** |
|  | Van Craenenbroeck K., Borroto-Escuela D.O., Romero-Fernandez W., Skieterska K., Rondou P., Lintermans B., Vanhoenacker P., Fuxe K., Ciruela F., Haegeman G. | Dopamine D4 receptor oligomerization - Contribution to receptor biogenesis | 2011 | FEBS Journal | 3 | NI | 12 | Laboratory of Eukaryotic Gene Expression and Signal Transduction (LEGEST), Ghent University Hospital, UZ Gent, Belgium; **Department of Neuroscience, Karolinska Institutet, Stockholm, Sweden;** Departament Patologia i TerapÌÄíöutica Experimental, IDIBELL-Universitat de Barcelona, L'Hospitalet de Llobregat, Barcelona, Spain; Center for Medical Genetics Ghent (CMGG), Ghent University Hospital, UZ Gent, Belgium; ActoGeniX, Technologiepark 4, Zwijnaarde, Belgium; Laboratory of Eukaryotic Gene Expression and Signal Transduction (LEGEST), Ghent University-UGent, KL Ledeganckstraat 35, 9000 Gent, Belgium |
|  | Borroto-Escuela D.O., Romero-Fernandez W., Tarakanov A.O., Ciruela F., Agnati L.F., Fuxe K. | On the existence of a possible A2A-D2-ë_- arrestin2 complex: A2A agonist modulation of D2 agonist-induced ë_-arrestin2 recruitment | 2011 | Journal of Molecular Biology | 4 | NI | 19 | **Department of Neuroscience, Karolinska Institutet, Retzius vÌ_g 8, 17177 Stockholm, Sweden;** St. Petersburg Institute for Informatics and Automation, Russian Academy of Sciences, 193167 Saint Petersburg, Russian Federation; Departament Patologia I TerapÌ¬utica Experimental, Facultat de Medicina, Universitat de Barcelona, 08907 Barcelona, Spain; IRCCS, 41100 Lido Venice, Italy |
|  | Borroto-Escuela D.O., Correia P.A., Romero-Fernandez W., Narvaez M., Fuxe K., Ciruela F., Garriga P. | Muscarinic receptor family interacting proteins: Role in receptor function | 2011 | Journal of Neuroscience Methods | 4 | NI | 10 | Centre de Biotecnologia Molecular, Department d'Enginyeria QuÌ_mica, Universitat PolitÌ¬cnica de Catalunya, Colom 1, 08222 Terrassa, Barcelona, Spain**; Department of Neuroscience, Karolinska Institutet, Retzius vÌ_g 8, 17177 Stockholm, Sweden;** W.M. Keck Center for Integrative Neuroscience, Department of Physiology, University of California, San Francisco, United States; Unitat de Farmacologia, Departament Patologia i TerapÌ¬utica Experimental, Facultat de Medicina, IDIBELL-Universitat de Barcelona, Barcelona, Spain |
|  | Borroto-Escuela D.O., Craenenbroeck K.V., Romero-Fernandez W., Guidolin D., Woods A.S., Rivera A., Haegeman G., Agnati L.F., Tarakanov A.O., Fuxe K. | Dopamine D2 and D4 receptor heteromerization and its allosteric receptor-receptor interactions | 2011 | Biochemical and Biophysical Research Communications | 7 | NI | 27 | **Department of Neuroscience, Karolinska Institutet, Stockholm, Sweden;** Laboratory of Eukaryotic Gene Expression and Signal Transduction, Ghent University, Belgium; Department of Human Anatomy and Physiology, University of Padova, Italy; NIDA-IRP, Structural Biology Unit, MD, United States; Department of Cell Biology, Faculty of Sciences, University of Malaga, Spain; IRCCS Lido Venice, Italy; Russian Academy of Sciences, St. Petersburg Institute for Informatics and Automation, Russian Federation |
|  | Borroto-Escuela D.O., Romero-Fernandez W., Tarakanov A.O., Gomez-Soler M., Corrales F., Marcellino D., Narvaez M., Frankowska M., Flajolet M., Heintz N., Agnati L.F., Ciruela F., Fuxe K. | Characterization of the A2AR-D2R interface: Focus on the role of the C-terminal tail and the transmembrane helices | 2010 | Biochemical and Biophysical Research Communications | 7 | NI | 17 | **Department of Neuroscience, Karolinska Institutet, Stockholm, Sweden;** Russian Academy of Sciences, St. Petersburg Institute for Informatics and Automation, Saint Petersburg, Russian Federation; Unitat de Farmacologia, Departament Patologia i TerapÌ¬utica Experimental, IDIBELL-Universitat de Barcelona, Spain; Centro de Neurociencias, La Habana, Cuba; Department of Physiology, School of Medicine, University of MÌÁlaga, Spain; Laboratory of Molecular and Cellular Neuroscience, The Rockefeller University, NY, United States; IRCCS Lido Venice, Italy |
|  | Borroto-Escuela D.O., Romero-Fernandez W., Tarakanov A.O., Marcellino D., Ciruela F., Agnati L.F., Fuxe K. | Dopamine D2 and 5-hydroxytryptamine 5-HT2A receptors assemble into functionally interacting heteromers | 2010 | Biochemical and Biophysical Research Communications | 4 | NI | 24 | **Department of Neuroscience, Karolinska Institutet, Retzius vÌ_g 8, 17177 Stockholm, Sweden;** Russian Academy of Sciences, St. Petersburg Institute for Informatics and Automation, Saint Petersburg, Russian Federation; Unitat de Farmacologia, Departament Patologia i TerapÌ¬utica Experimental, Facultat de Medicina, IDIBELL-Universitat de Barcelona, Barcelona, Spain; IRCCS Lido Venice, Italy |
|  | Ciruela F., Vallano A., Arnau J.M., Snchez S., Borroto-Escuela D.O., Agnati L.F., Fuxe K., Fernndez-Dueas V. | G protein-coupled receptor oligomerization for what | 2010 | Journal of Receptors and Signal Transduction | 3 | NI | 11 | Departament de Patologia i Teraputica Experimental, Facultat de Medicina, Universitat de Barcelona, 08907 Barcelona, Spain; IRCCS San Camillo, Lido Venezia, Italy**; Department of Neuroscience, Karolinska Institutet, Stockholm, Sweden** |
|  | Fuxe K., Marcellino D., Borroto-Escuela D.O., Frankowska M., Ferraro L., Guidolin D., Ciruela F., Agnati L.F. | The changing world of G protein-coupled receptors: From monomers to dimers and receptor mosaics with allosteric receptorreceptor interactions | 2010 | Journal of Receptors and Signal Transduction | 4 | NI | 33 | **Department of Neuroscience, Karolinska Institutet, Stockholm, SE-171 77, Sweden;** Department of Experimental and Clinical Medicine, Section of Pharmacology and BioPharmaNet TransLab, University of Ferrara, Ferrara, Italy; Department of Human Anatomy and Physiology, University of Padova, Padova, Italy; Departament de Patologia i Teraputica Experimental, Facultat de Medicina, Universitat de Barcelona, Barcelona, Spain; IRCCS San Camillo, Lido Venezia, Italy |
|  | Borroto-Escuela D.O., Garcia-Negredo G., Garriga P., Fuxe K., Ciruela F. | The M5 muscarinic acetylcholine receptor third intracellular loop regulates receptor function and oligomerization | 2010 | Biochimica et Biophysica Acta - Molecular Cell Research | 3 | NI | 14 | Centre de Biotecnologia Molecular, Department d'Enginyeria QuÌ_mica, Universitat PolitÌ¬cnica de Catalunya, Terrassa, 08222 Barcelona, Spain; Unitat de Farmacologia, Departament Patologia i TerapÌ¬utica Experimental, Facultat de Medicina, IDIBELL-Universitat de Barcelona, L'Hospitalet de Llobregat, 08907 Barcelona, Spain**; Neuroscience Department, Karolinska Institutet, Stockholm, Sweden** |
|  | Fuxe K., Marcellino D., Borroto-Escuela D.O., Guescini M., Fernandez-Duenas V., Tanganelli S., Rivera A., Ciruela F., Agnati L.F. | Adenosine-dopamine interactions in the pathophysiology and treatment of cns disorders | 2010 | CNS Neuroscience and Therapeutics | 6 | NI | 40 | **Department of Neuroscience, Karolinska Institutet, Retzius vÌ_g 8, Stockholm, Sweden;** Department of Biomolecular Sciences, University of Urbino Carlo Bo, Italy; Departament Patologia i TerapíÛeutica Experimental, Facultat de Medicina, Universitat de Barcelona, L Hospitalet de Llobregat, Barcelona, Spain; Department of Clinical and Experimental Medicine, Section of Pharmacology, University of Ferrara, Ferrara, Italy; Department of Cell Biology, University of Malaga, Malaga, Spain; Department of Biomedical Sciences, University of Modena, Modena, and IRCCS Lido, Venice, Italy |
|  | Borroto-Escuela D.O., Correia P.A., Perez Alea M., Narvaez M., Garriga P., Fuxe K., Ciruela F. | Impaired M3 Muscarinic acetylcholine receptor signal transduction through blockade of binding of multiple proteins to its third intracellular loop | 2010 | Cellular Physiology and Biochemistry | 5 | Ni | 13 | **Neuroscience Department, Karolinska Institutet, Retzius vÌ_g 8, Stockholm, Sweden;** W.M. Keck Centre for Integrative Neuroscience, Department of Physiology, University of California, San Francisco, United States; Centre for Molecular Biotechnology, Department of Chemical Engineering, Technical University of Catalonia, Barcelona, Spain; Department of Physiology, School of Medicine, University of MÌÁlaga, United States; Department of Pathology and Experimental Therapeutic, School of Medicine, IDIBELL-University of Barcelona, Barcelona, Spain |
|  | Borroto-Escuela D.O., Marcellino D., Narvaez M., Flajolet M., Heintz N., Agnati L., Ciruela F., Fuxe K. | A serine point mutation in the adenosine A2AR C-terminal tail reduces receptor heteromerization and allosteric modulation of the dopamine D2R | 2010 | Biochemical and Biophysical Research Communications | 5 | NI | 17 | **Department of Neuroscience, Karolinska Institutet, Stockholm, Sweden;** Department of Physiology, School of Medicine, University of MÌÁlaga, Spain; Laboratory of Molecular and Cellular Neuroscience, The Rockefeller University, NY, United States; Department of Biochemical Sciences, University of Modena and IRCCS Lido, Venice, Italy; Unitat de Farmacologia, Departament Patologia i TerapÌ¬utica Experimental, Facultat de Medicina, Barcelona, Spain |
|  | Borroto-Escuela D.O., Narvaez M., Marcellino D., Parrado C., Narvaez J.A., Tarakanov A.O., Agnati L.F., Diaz-Cabiale Z., Fuxe K. | Galanin receptor-1 modulates 5-hydroxtryptamine-1A signaling via heterodimerization | 2010 | Biochemical and Biophysical Research Communications | 4 | NI | 28 | **Department of Neuroscience, Karolinska Institutet. Retzius vag 8, 17177 Stockholm, Sweden;** Department of Physiology, School of Medicine, University Malaga. Campus Teatinos s/n, 29071 MÌÁlaga, Spain; Department of Histology, School of Medicine, University Malaga. Campus Teatinos s/n, 29071 MÌÁlaga, Spain; Russian Academy of Sciences, St. Petersburg Institute for Informatics and Automation, Saint Petersburg, Russian Federation; Department of Biomedical Sciences, University of Modena and IRCCS Lido Venice, Italy |
|  | Borroto Escuela D.O., Alea M.P., Fernandez W.R., Gil D.B. | Vectors and P64k gene targeting for tandem affinity purification in Neisseria meningitidis | 2006 | Journal of Microbiological Methods | 1 | N | 5 | **Universidad Politecnica de Catalunya, Ingenieria Quimica, Colom 1. EUETIT. TR1, 08222 Terrassa, Spain** |

**SUMMARY**

|  |  |  |  |  | Types of Collaboration | Nr articles in Europe |
| --- | --- | --- | --- | --- | --- | --- |
|  |  |  |  |  | N | 2 |
|  | Data analysis (source Scopus BiblioCRiE dataset): | |  |  | NN | 0 |
|  | Year 1 (first publication): 2006 | |  |  | NI | 56 |
|  | Year 5 (2010) one article in collaboration with Cuba | |  |  | NNI | 1 |
|  | Year 8 (2013) two articles in collaboration with Cuba | |  |  |  | 59 |
|  | Total number of collaborating institutions: 34 generating 57 collaborative articles (NI,NNI) with 210 links or collaborations | |  |  |  | |
|  |  |  |  | Publishing with Cuban affiliation | Pc | 0 |
|  | Only one Cuban institution, 3 articles, 3 links | |  | Publishing with European affiliation | PE | 59 |
|  | This CRiE move from Spain (PhD) to Sweden (Postdoc) | |  | Collaboration with Cuba from European institutions | Cc | 3 |
|  |  |  |  | Total collaborative articles from Europe | CE | 57 |
