# Supplementary material for: Scientific collaboration of Cuban researchers working in Europe: understanding relations between origin and destination countries
Source: Scientometrics. 2018 Aug 20;117(2):745–69. doi: 10.1007/s11192-018-2888-2 (PMC6280978; doi:10.1007/s11192-018-2888-2)
Supplement: Supplementary file 2 — Aggregate values of PC, PE, CE, CC of 107 CRiE according to their nexus with Cuba between 1995 and 2014. (DOCX 134 kb) [file 11192_2018_2888_MOESM2_ESM.docx]

**APPENDIX B CRiE nexus CUBA**

CRiE were classified according to their nexus with Cuban institutions shown in their publications

Number of publications having Cuban affiliation (Pc) or European affiliation (Pe)

Number of collaborative publications within Pe (Ce) of which Cc are with Cuban institutions

|  | CRiE never published with Cuban affiliation (Pc=0) neither had collaborative articles with Cuban institutions (Cc=0) | | | | |  | CRiE never published with Cuban affiliation (Pc=0), but had collaborative articles with Cuban institutions (Cc>0) | | | | |  | CRiE have published with Cuban affiliation (Pc>0), but never had collaborative articles with Cuban institutions (Cc=0) | | | | |  | CRiE have published with Cuban affiliation (Pc>0) and also have collaborative articles with Cuban institutions (Cc>0) | | | | |
| --- | --- | --- | --- | --- | --- | --- | --- | --- | --- | --- | --- | --- | --- | --- | --- | --- | --- | --- | --- | --- | --- | --- | --- |
|  |  |  |  |  |  |  |  |  |  |  |  |  |  |  |  |  |  |  |  |  |  |  |  |
|  | **P2C2** | Pc Exp Cuba | Pe Exp Europe | Ce | Cc |  | **P2C1** | Pc Exp Cuba | Pe Exp Europe | Ce | Cc |  | **P1C2** | Pc Exp Cuba | Pe Exp Europe | Ce | Cc |  | **P1C1** | Pc Exp Cuba | Pe Exp Europe | Ce | Cc |
|  | 201-01 | 0 | 7 | 6 | 0 |  | 201-02 | 0 | 2 | 2 | 1 |  | 202-02 | 8 | 12 | 11 | 0 |  | 201-03 | 8 | 11 | 8 | 6 |
|  | 201-04 | 0 | 17 | 7 | 0 |  | 201-15 | 0 | 24 | 18 | 2 |  | 202-06 | 1 | 1 | 1 | 0 |  | 201-06 | 4 | 43 | 23 | 5 |
|  | 201-05 | 0 | 3 | 3 | 0 |  | 202-09 | 0 | 3 | 2 | 2 |  | 203-05 | 5 | 6 | 3 | 0 |  | 201-09 | 10 | 3 | 3 | 1 |
|  | 201-07 | 0 | 37 | 34 | 0 |  | 203-13 | 0 | 3 | 2 | 1 |  | 203-09 | 1 | 9 | 9 | 0 |  | 201-10 | 3 | 14 | 7 | 3 |
|  | 201-08 | 0 | 38 | 20 | 0 |  | 207-02 | 0 | 8 | 6 | 4 |  | 203-11 | 4 | 9 | 8 | 0 |  | 202-03 | 24 | 3 | 2 | 2 |
|  | 202-01 | 0 | 17 | 16 | 0 |  | 207-07 | 0 | 19 | 18 | 2 |  | 203-12 | 4 | 2 | 1 | 0 |  | 202-05 | 19 | 3 | 3 | 2 |
|  | 203-02 | 0 | 8 | 5 | 0 |  | 207-11 | 0 | 43 | 30 | 1 |  | 204-01 | 3 | 28 | 24 | 0 |  | 203-01 | 21 | 17 | 14 | 5 |
|  | 203-06 | 0 | 4 | 2 | 0 |  | 207-14 | 0 | 2 | 2 | 1 |  | 204-03 | 2 | 1 | 1 | 0 |  | 203-03 | 1 | 32 | 26 | 7 |
|  | 203-08 | 0 | 2 | 2 | 0 |  | 207-19 | 0 | 5 | 4 | 2 |  | 205-02 | 4 | 9 | 4 | 0 |  | 203-04 | 2 | 51 | 43 | 6 |
|  | 205-01 | 0 | 13 | 5 | 0 |  | 207-27 | 0 | 2 | 2 | 1 |  | 205-04 | 2 | 1 | 1 | 0 |  | 203-07 | 4 | 2 | 2 | 1 |
|  | 205-06 | 0 | 3 | 0 | 0 |  | 207-28 | 0 | 2 | 2 | 1 |  | 205-05 | 12 | 3 | 3 | 0 |  | 204-02 | 1 | 27 | 22 | 6 |
|  | 207-10 | 0 | 7 | 3 | 0 |  | 207-35 | 0 | 20 | 6 | 1 |  | 205-07 | 2 | 15 | 9 | 0 |  | 205-03 | 4 | 5 | 5 | 1 |
|  | 207-15 | 0 | 39 | 27 | 0 |  | 209-01 | 0 | 59 | 57 | 3 |  | 205-08 | 2 | 21 | 7 | 0 |  | 205-10 | 5 | 3 | 1 | 1 |
|  | 207-17 | 0 | 2 | 0 | 0 |  | 209-03 | 0 | 28 | 28 | 2 |  | 205-09 | 2 | 7 | 5 | 0 |  | 206-01 | 10 | 21 | 21 | 21 |
|  | 207-23 | 0 | 103 | 54 | 0 |  | 210-04 | 0 | 31 | 24 | 1 |  | 207-04 | 4 | 2 | 2 | 0 |  | 207-01 | 1 | 3 | 3 | 1 |
|  | 207-36 | 0 | 5 | 1 | 0 |  | SUM | 0 | 251 | 203 | 25 |  | 207-13 | 8 | 5 | 3 | 0 |  | 207-03 | 3 | 14 | 13 | 1 |
|  | 207-37 | 0 | 3 | 3 | 0 |  | Mean | 0.0 | 16.7 | 13.5 | 1.7 |  | 207-25 | 2 | 5 | 3 | 0 |  | 207-05 | 30 | 11 | 10 | 8 |
|  | 208-01 | 0 | 15 | 7 | 0 |  | Std Dev | 0.0 | 17.6 | 15.9 | 0.9 |  | 207-26 | 1 | 2 | 2 | 0 |  | 207-06 | 4 | 11 | 10 | 1 |
|  | 208-04 | 0 | 1 | 1 | 0 |  | n=15 |  |  |  |  |  | 207-39 | 3 | 17 | 15 | 0 |  | 207-08 | 1 | 7 | 7 | 4 |
|  | 210-09 | 0 | 192 | 173 | 0 |  |  |  |  |  |  |  | 208-02 | 8 | 1 | 1 | 0 |  | 207-09 | 20 | 8 | 2 | 1 |
|  | 210-11 | 0 | 13 | 11 | 0 |  |  |  |  |  |  |  | 208-03 | 1 | 13 | 5 | 0 |  | 207-12 | 5 | 52 | 28 | 1 |
|  | 210-12 | 0 | 17 | 17 | 0 |  |  |  |  |  |  |  | 209-02 | 3 | 6 | 2 | 0 |  | 207-16 | 2 | 6 | 6 | 1 |
|  | 210-18 | 0 | 12 | 3 | 0 |  |  |  |  |  |  |  | 210-05 | 2 | 2 | 1 | 0 |  | 207-21 | 20 | 11 | 10 | 6 |
|  | SUM | 0 | 558 | 400 | 0 |  |  |  |  |  |  |  | 210-10 | 1 | 2 | 2 | 0 |  | 207-22 | 77 | 27 | 19 | 5 |
|  | Mean | 0 | 24.3 | 17.4 | 0 |  |  |  |  |  |  |  | 210-14 | 6 | 2 | 0 | 0 |  | 207-24 | 7 | 8 | 2 | 1 |
|  | Std Dev | 0 | 42.7 | 36.4 | 0 |  |  |  |  |  |  |  | 210-20 | 6 | 14 | 8 | 0 |  | 207-29 | 3 | 21 | 17 | 6 |
|  | n=23 |  |  |  |  |  |  |  |  |  |  |  | 213-01 | 2 | 12 | 10 | 0 |  | 207-30 | 1 | 7 | 7 | 6 |
|  |  |  |  |  |  |  |  |  |  |  |  |  | SUM | 99 | 207 | 141 | 0 |  | 207-31 | 7 | 8 | 4 | 1 |
|  |  |  |  |  |  |  |  |  |  |  |  |  | Mean | 3.7 | 7.7 | 5.2 | 0.0 |  | 207-32 | 3 | 7 | 5 | 1 |
|  |  |  |  |  |  |  |  |  |  |  |  |  | Std Dev | 2.8 | 7.0 | 5.4 | 0.0 |  | 207-33 | 17 | 130 | 100 | 36 |
|  |  |  |  |  |  |  |  |  |  |  |  |  | n=27 |  |  |  |  |  | 207-40 | 1 | 5 | 3 | 1 |
|  |  |  |  |  |  |  |  |  |  |  |  |  |  |  |  |  |  |  | 209-04 | 28 | 8 | 6 | 7 |
| Code: country-researcher. 201= Belgium, 202= France, 203= Germany, 204= Finland, 205= Italy, 206= Portugal, 207= Spain, 208= Switzerland, 209=Sweden, 210= United Kingdom, 213= Netherlands, 216= Denmark, 225= Luxemburg | | | | | | | | | | |  |  |  |  |  |  |  |  | 210-01 | 1 | 22 | 16 | 1 |
|  |  |  |  |  |  |  |  |  |  |  |  |  |  |  |  |  |  |  | 210-02 | 3 | 41 | 33 | 3 |
|  |  |  |  |  |  |  |  |  |  |  |  |  |  |  |  |  |  |  | 210-03 | 25 | 132 | 68 | 9 |
|  |  |  |  |  |  |  |  |  |  |  |  |  |  |  |  |  |  |  | 210-06 | 1 | 2 | 2 | 1 |
|  |  |  |  |  |  |  |  |  |  |  |  |  |  |  |  |  |  |  | 210-13 | 21 | 3 | 3 | 1 |
|  |  |  |  |  |  |  |  |  |  |  |  |  |  |  |  |  |  |  | 210-15 | 5 | 29 | 22 | 2 |
|  |  |  |  |  |  |  |  |  |  |  |  |  |  |  |  |  |  |  | 210-17 | 9 | 6 | 2 | 1 |
|  |  |  |  |  |  |  |  |  |  |  |  |  |  |  |  |  |  |  | 210-19 | 2 | 18 | 14 | 2 |
|  |  |  |  |  |  |  |  |  |  |  |  |  |  |  |  |  |  |  | 216-01 | 1 | 2 | 2 | 1 |
|  |  |  |  |  |  |  |  |  |  |  |  |  |  |  |  |  |  |  | 228-01 | 2 | 13 | 10 | 2 |
|  |  |  |  |  |  |  |  |  |  |  |  |  |  |  |  |  |  |  | SUM | 416 | 847 | 604 | 178 |
|  |  |  |  |  |  |  |  |  |  |  |  |  |  |  |  |  |  |  | Mean | 9.9 | 20.2 | 14.4 | 4.2 |
|  |  |  |  |  |  |  |  |  |  |  |  |  |  |  |  |  |  |  | Std Dev | 13.7 | 28.4 | 18.8 | 6.2 |
|  |  |  |  |  |  |  |  |  |  |  |  |  |  |  |  |  |  |  | n=42 |  |  |  |  |
