# Supplementary material for: Scientific collaboration of Cuban researchers working in Europe: understanding relations between origin and destination countries
Source: Scientometrics. 2018 Aug 20;117(2):745–69. doi: 10.1007/s11192-018-2888-2 (PMC6280978; doi:10.1007/s11192-018-2888-2)
Supplement: Supplementary file 3 — CRiE 209-01 segment matrix. Using Scopus institutional addresses (sheet 1) a matrix (sheet 2) of three columns is created as Type I institution (CRiE affiliation), type II institution (collaborating) and number of their collaboration or links. The aggregate data of 107 CRiE is transformed in one symmetric matrix using ad-hoc program (Marcet García et al. 2016). (DOCX 105 kb) [file 11192_2018_2888_MOESM3_ESM.docx]

APPENDIX C: CRiE 209-01- Segment of the general matrix (one out 107 CRiE). Collaborating institutions were taken from Appendix A.

| **Author Scous ID** | **Type I institution: Name CRiE institution** | **Code CRiE institution** | **Type II institution: Name collaborating institution** | **Code collaborating institution** | **All links** | **Cuban links** |  |  |  | **code CRiE institution** | **Code collaborating institution** | **All links** |
| --- | --- | --- | --- | --- | --- | --- | --- | --- | --- | --- | --- | --- |
| **Scopus ID: 35723721100 CRiE code: 209-01** | Department of Neuroscience, **Karolinska Institutet.** Retzius vag 8, 17177 Stockholm, Sweden | 209005 | Department of Physiology, School of Medicine, **University Malaga**. Campus Teatinos s/n, 29071 MÌÁlaga, Spain | 207022 | 25 | 0 |  |  |  | 209005 | 207022 | 25 |
|  |  |  | Russian Academy of Sciences, **St. Petersburg Institute for Informatics and Automation,** Russian Academy of Science, Saint Petersburg, Russian Federation; | 220001 | 23 | 0 |  |  |  | 209005 | 220001 | 23 |
|  |  |  | Department of Biomedical Sciences, **University of Modena, Italy** | 205057 | 10 | 0 |  |  |  | 209005 | 205057 | 10 |
|  |  |  | Laboratory of Molecular and Cellular Neuroscience, **The Rockefeller University**, NY, United States; | 302067 | 3 | 0 |  |  |  | 209005 | 302067 | 3 |
|  |  |  | Unitat de Farmacologia, Departament Patologia i TerapÌ¬utica Experimental, Facultat de Medicina, **Universidad de Barcelon**a, Spain | 207023 | 28 | 0 |  |  |  | 209005 | 207023 | 28 |
|  |  |  | W.M. Keck Centre for Integrative Neuroscience, Department of Physiology, **University of California**, San Francisco, United States; | 302005 | 3 | 0 |  |  |  | 209005 | 302005 | 3 |
|  |  |  | Centre for Molecular Biotechnology, Department of Chemical Engineering, Departament d'Enginyeria QuÌ_mica, **Universitat PolitÌ¬cnica de Catalunya**, Terrassa 08222, Spain **Technical University of Catalonia**, Barcelona, Spain | 207021 | 13 | 0 |  |  |  | 209005 | 207021 | 13 |
|  |  |  | Department of Biomolecular Sciences, **University of Urbino Carlo Bo**, Italy; | 205067 | 4 | 0 |  |  |  | 209005 | 205067 | 4 |
|  |  |  | Department of Clinical and Experimental Medicine, Section of Pharmacology, **University of Ferrara,** Ferrara, Italy; | 205006 | 9 | 0 |  |  |  | 209005 | 205006 | 9 |
|  |  |  | Department of Human Anatomy and Physiology, **University of Padova**, Italy; | 205064 | 10 | 0 |  |  |  | 209005 | 205064 | 10 |
|  |  |  | **IRCCS** San Camillo, Lido Venezia, Italy; | 205051 | 31 | 0 |  |  |  | 209005 | 205051 | 26 |
|  |  |  | **Centro de Neurociencias (CNC)**, La Habana, Cuba; | 101025 | 3 | 3 |  |  |  | 209005 | 101025 | 3 |
|  |  |  | Laboratory of Eukaryotic Gene Expression and Signal Transduction, **Ghent University**, Belgium | 201015 | 6 | 0 |  |  |  | 209005 | 201015 | 6 |
|  |  |  | NIDA **[National Institute on Drug Abuse**]-IRP, Structural Biology Unit, MD, United States | 302071 | 2 | 0 |  |  |  | 209005 | 302071 | 2 |
|  |  |  | Division of Human Physiology, Laboratory of Molecular Neurobiology, **University of Palermo,** Corso Tukory 129, 90134 Palermo, Italy; | 205005 | 3 | 0 |  | 🡺 |  | 209005 | 205005 | 3 |
|  |  |  | School of Life and Health, **Aston University**, Birmingham, United Kingdom | 210010 | 4 | 0 |  |  |  | 209005 | 210010 | 4 |
|  |  |  | **Institut d'Investigacions BiomÌ¬diques de Barcelona (IIBB),** Barcelona, Spain | 207024 | 1 | 0 |  |  |  | 209005 | 207024 | 1 |
|  |  |  | Laboratory of Drug Addiction Pharmacology, Department of Pharmacology, **Institute of Pharmacology Polish Academy of Sciences,** KrakÌ_w, Poland; | 214008 | 4 | 0 |  |  |  | 209005 | 214008 | 4 |
|  |  |  | Departamento de BioquÌ_mica Centro de CiÌ»ncias BiolÌ_gicas, **Universidade Federal de Santa Catarina**, Florianopolis, SC 88040-900, Brazil | 401013 | 2 | 0 |  |  |  | 209005 | 401013 | 2 |
|  |  |  | School of Anatomical Sciences, Faculty of Health Sciences, **University of the Witwatersrand**, 7 York Road, Parktown, 2193, South Africa; | 501003 | 2 | 0 |  |  |  | 209005 | 501003 | 2 |
|  |  |  | School of Biological Sciences, **University of Bristol,** Bristol, United Kingdom; | 210010 | 1 | 0 |  |  |  | 209005 | 210010 | 1 |
|  |  |  | Proteomics Unit, IRCCS Centro S. Giovanni di Dio-Fatebenefratelli, Brescia, Italy; | 205068 | 1 | 0 |  |  |  | 209005 | 205068 | 1 |
|  |  |  | Laboratory of Bioorganic Chemistry, National Institute of Diabetes and Digestive and Kidney Diseases, **National Institutes of Health, Bethesda,** MD, United States | 302009 | 1 | 0 |  |  |  | 209005 | 302009 | 1 |
|  |  |  | Human Brain Tissue Bank, **Semmelweis University**, Budapest, Hungary | 211008 | 3 | 0 |  |  |  | 209005 | 211008 | 3 |
|  |  |  | Institute of Acupuncture and Moxibustion, **China Academy of Chinese Medical Sciences**, Beijing 100700, China | 602011 | 1 | 0 |  |  |  | 209005 | 602011 | 1 |
|  |  |  | **IIIA-CSIC,** Artificial Intelligence Research Institute, Spanish National Research Council, 08193 Barcelona, Spain | 207076 | 2 | 0 |  |  |  | 209005 | 207076 | 2 |
|  |  |  | Department of Physiology, Faculty of Medicine, **University of Tartu,** Estonia | 226001 | 3 | 0 |  |  |  | 209005 | 226001 | 3 |
|  |  |  | Department of Biophysic, Instituto de FisiologÌ_a Celular, **Universidad Nacional AutÌ_noma de MÌ©xico**, MÌ©xico, DF, Mexico; | 104002 | 2 | 0 |  |  |  | 209005 | 104002 | 2 |
|  |  |  | Department of Pharmacy and Center of Excellence for Biomedical Research (CEBR), **University of Genova,** Genoa, Italy | 205040 | 1 | 0 |  |  |  | 209005 | 205040 | 1 |
|  |  |  | Department of Earth, Life and Environmental Sciences, Section of Physiology, **Campus Scientifico 'Enrico Mattei**', Urbino, Italy | 205013 | 4 | 0 |  |  |  | 209005 | 205013 | 4 |
|  |  |  | Department of Neuroscience, **Biodonostia Institute [Ikerbasque],** 20014 San SebastiÌÁn, Spain; | 207068 | 1 | 0 |  |  |  | 209005 | 207068 | 1 |
|  |  |  | **Technical University of Ambato**, Ecuador; | 405002 | 1 | 0 |  |  |  | 209005 | 405002 | 1 |
|  |  |  | Department of Biochemistry and Biophysics, **Stockholm University**, Stockholm, Sweden | 209011 | 2 | 0 |  |  |  | 209005 | 209011 | 2 |
|  |  |  | Unitat de Farmacologia, Departament de Patologia i TerapÌ¬utica Experimental, **L'Hospitalet del Llobregat Barcelona**, PavellÌ_ de Govern Av. Feixa Llarga s/n 08907, Spain; | 207135 | 1 | 0 |  |  |  | 209005 | 207135 | 1 |
|  |  |  |  |  | 210 | 3 |  |  |  |  |  |  |
