# Supplementary material for: Scientific collaboration of Cuban researchers working in Europe: understanding relations between origin and destination countries
Source: Scientometrics. 2018 Aug 20;117(2):745–69. doi: 10.1007/s11192-018-2888-2 (PMC6280978; doi:10.1007/s11192-018-2888-2)
Supplement: Supplementary file 4 — CRiE vs. CRiC. Cuban researchers in Europe (106) were compared to top Cuban researchers (100) from 34 Cuban institutions in terms of their Seniority (S) and Productivity (P). (DOCX 356 kb) [file 11192_2018_2888_MOESM4_ESM.docx]

APPENDIX D. Cuban researchers in Cuba (CRiC) versus CRiE

The information shown here is taken from of the Doctoral Thesis of Miriam Palacios-Callender (2016, chapters 4 and 6) and it is mainly to provide additional information regarding the group CRiC mentioned in this article.

In *Mobility, migration and networking of Cubans working in European science and technology: building capacity through transnational knowledge networks*. Doctoral Thesis. School of Computing and Engineering, University of West London, Ealing, United Kingdom. Electronic theses online service (Ethos) at the British Library: I**SNI:** [0000 0004 6056 9864](http://isni.org/isni/0000000460569864) <http://ethos.bl.uk/OrderDetails.do?did=1&uin=uk.bl.ethos.704577>

Experimental design comparing CRiE with top performers CRiC

Selecting the group of Cuban Researchers in Cuba (CRiC): The group of Cuban Researchers working in Cuban institutions was obtained by searching in Scopus first for <Affiliation> retrieving 99 Cuban institutions of which 4 were counted twice (due to names in Spanish and English of the same institution) and a non-Cuban institution (US-Cuba Project, Global Options Group Inc., New York, searching 28th October 2014). For each institution Scopus listed the number of authors and documents (articles). Choosing <authors> gives the list of researchers’ names by descending order according to their number of publications. To ensure representation of all institutions a cut-off of number of publications per researcher was set to more than 47 articles per author. Those who stopped publishing in the last two years were excluded.

The search in *Scopus* only showed 94 Cuban institutions out of 318 reported in a study about the scientific outputs of Cuban institutions in which Scopus was also used (Arencibia Jorge et al., 2013). In the case of researchers’ names searching was also carried out using a combination of their first and second surnames. Two researchers were therefore eliminated due to merging authors with common name and surnames, inasmuch the fields and places of publications did not match. The following table shows the composition of CRiC per their research institution. One hundred researchers from thirty-four Cuban institutions were included in the study. The original work in the doctoral thesis includes an appendix with the code and name of institutions.

Relevant results for this article:

*Gender, place and year of higher education attainment and post graduate studies*

The study sample did not reflect the Cuban gender composition in research institutions with average 53.8% female researchers between 2005 and 2010 (ONE, 2014), although this percentage refers to the total composition and not specifically to active researchers. Female CRiE were only 34% (36 female out of 106 active researchers). Female CRiC were 25% (30 out of 120 CRiC) when selecting researchers with more than 48 publications in the period working in 14 different institutions out of 94 listed through the *Scopus* search (Table 4.3). The bigger group of female CRiE attained their HE in the years from 1995 to 1999 and for the male active researchers during the years 1995 to 2009, as shown in figure 6.1. Those years in which larger group of CRiE graduated from Cuban universities correspond to researchers between 30 and 40 years old, which is the age related group of the Cuban population with the highest proportion of migration (Sorolla Fernádez, 2013). Assuming those researchers did not have any interruption during their education, 67% (21 out of 33) of the females and 77% (46 out of 60) of the males were younger than 40 years old.

CRiE graduated from academic institutions in Havana represent 89% of those who stated their places of HE graduation in the public domain (98 researchers, Table 6.3). This is in agreement with previous findings of Havana as a sender city of tertiary educated (Martín Fernández, 2007) migrants. Other factors behind this high representation of Havana in the CRiE sample might be the density of HE institutions in Havana (seven out of twenty one universities in the Ministry of Higher Education are in Havana) holding more students than the rest of the country, and the quality of education attained in those institutions with high standards, allowing the graduates to secure places in European universities and research institutions. The quality of education in this case refers to the possibility of students being part of an environment with a long tradition of publishing in scientific journals as it is the case of University of Havana (Palacios-Callender et al., 2016) and with strong national and international collaboration.

Figure 6.1 CRiE: Gender and year of HE attained

Another characteristic of the CRiE is the low proportion of engineers with only 17% involved in research. This could be due to the filter applied for active researchers; publication pattern in science differs from engineering that might have patents as output rather than publications in scientific journals.

*Cuban scientists in Europe (CRiE) and in Cuba (CRiC)*

The bibliometric indicators used to investigate the Cuban researchers were productivity and seniority. Productivity refers to the total number of publications per researcher and seniority refers to the length of time in years since the researcher published for the first time. They also refer to a specific period (1995-2014) and places (Cuba or Europe).

This study does not aim to establish a quantitative comparison between both groups, but to have an indication of where CRiE stand in relation to the best performers in Cuba. Among the reasons why the quantitative comparison is not valid here are the differences of age groups, the type of research as applied or basic research, different fields of research within and between both groups, etc.

Researchers in some academic institutions have teaching as a priority compared with those in research institution and therefore publishing less. Group leaders also get the benefit of the contribution of the team members to science. The high values of the standard deviations in both groups for the number of articles indicates the heterogeneity of both samples due to different areas and fields of research as well as the stages in their careers. Table 6.6 shows means and standard deviations for seniority and productivity of CRiE and CRiC according to time and places (Cuba versus Europe).

Table 6.6 Means and standard deviations of indicators for Cuban Researchers

The total publishing years of CRiE is less than half of those for CRiC even for the period of study (1995-2014), indicating that CRiE are younger researchers as seen in the previous section 6.2.2 (Figure 6.1), publishing a third of the CRiC group in general and for the period of study. The gap is also seen even counting CRiE publications while working in Cuba (P_(20)_). The difference is less dramatic in the last five years as CRiE are gaining in seniority.

The standard deviations for *Productivity* are high in all groups as researchers come from fields of research with different patterns of publications. Although there is not a CRiE - CRiC matching for each researcher the multidisciplinary subjects in both groups are similar, reflecting the strength of Cuban science (biotechnology, bioinformatics, theoretical chemistry, physics, immunology, etc.). Choosing CRiC from 34 Cuban institutions might have contributed to improving the groups’ similarities in terms of fields of research.

Conclusion:

Gender composition of the CRiE sample corresponds to 34% female versus 25% in the reference group of CRiC. CRiE are younger researchers shown by both the number of years publishing (11 versus 25 years for CRiC) and the high proportion of them graduated after 1995, indicating that the 77% are younger than 40 years old.

The productivity gap between groups should not be interpreted as a difference in the performance pattern between Cuba and Europe, but as a consequence of the generational gap between top researchers in Cuba and those young researchers furthering their education in Europe.
